# Genome-wide identification of a novel Na^+^ transporter from *Bienertia sinuspersici* and overexpression of *BsHKT1;2* improved salt tolerance in *Brassica rapa*

**DOI:** 10.3389/fpls.2023.1302315

**Published:** 2023-12-12

**Authors:** Vadivelmurugan Irulappan, Hyun Woo Park, Sang-Yun Han, Myung-Hee Kim, Jung Sun Kim

**Affiliations:** Genomics Division, Department of Agricultural Bio-Resources, National Institute of Agricultural Sciences, Jeonju, Republic of Korea

**Keywords:** *Bienertia sinuspersici*, *Brassica rapa*, high-affinity K^+^ transporter (HKT), salt stress, abiotic stress, Na^+^/H^+^ exchangers (NHX), glycophyte, halophyte

## Abstract

Salt stress is an ever-increasing stressor that affects both plants and humans. Therefore, developing strategies to limit the undesirable effects of salt stress is essential. Sodium ion exclusion is well known for its efficient salt-tolerance mechanism. The High-affinity K^+^ Transporter (HKT) excludes excess Na^+^ from the transpiration stream. This study identified and characterized the HKT protein family in *Bienertia sinuspersici*, a single-cell C_4_ plant. The *HKT* and *Salt Overly Sensitive 1 (SOS1)* expression levels were examined in *B. sinuspersici* and *Arabidopsis thaliana* leaves under four different salt stress conditions: 0, 100, 200, and 300 mM NaCl. Furthermore, *BsHKT1;2* was cloned, thereby producing stable transgenic *Brassica rapa*. Our results showed that, compared to *A. thaliana* as a glycophyte, the HKT family is expanded in *B. sinuspersici* as a halophyte with three paralogs. The phylogenetic analysis revealed three paralogs belonging to the HKT subfamily I. Out of three copies, the expression of *BsHKT1;2* was higher in *Bienertia* under control and salt stress conditions than in *A. thaliana*. Stable transgenic plants overexpressing *35S::BsHKT1;2* showed higher salt tolerance than non-transgenic plants. Higher biomass and longer roots were observed in the transgenic plants under salt stress than in non-transgenic plants. This study demonstrates the evolutionary and functional differences in HKT proteins between glycophytes and halophytes and associates the role of *BsHKT1;2* in imparting salt tolerance and productivity.

## 1 Introduction

Salt stress, the second most important abiotic stress, is a global concern that challenges food security by affecting crop growth and yield factors (Rengasamy, 2006; Munns and Tester, 2008; Zhu, 2016). Salt stress affects more than 6% of the total land area worldwide and 20% of irrigated land, restraining global food production (Rengasamy, 2006; Munns and Tester, 2008). The Mediterranean, semi-arid, and arid areas have experienced severe salinity effects. In South Korea, reclaimed tidelands (RTLs) are used to cultivate crops such as rice. However, cultivating crops such as *Brassica* in the RTL is still impossible due to high salinity and poor soil physical properties (Hong et al., 2020; Lim et al., 2020). Soil containing 40 mM NaCl potentially affects crop yield by inducing osmotic and ionic stress (Flowers and Colmer, 2008; Huang et al., 2008; Munns and Tester, 2008). In plants, three major mechanisms have been demonstrated to cope with salinity stress: Na^+^ extrusion into the soil involving salt overly sensitive 1 (SOS1) (Shi et al., 2000; Shi et al., 2003; Zhu, 2016); Na^+^ ion exclusion from the xylem stream involving high-affinity K^+^ transporter 1 (HKT1) (Munns and Tester, 2008; Møller et al., 2009; Munns et al., 2012); and Na^+^ sequestration into vacuoles involving Na^+^/H^+^ exchangers 1-4 (NHX1-4) antiporters (Zhang et al., 2019; Van Zelm et al., 2020). In addition to HKT and NHX transporters, studies have demonstrated that the high-affinity K^+^ (HAK) transporter located in the plasma membrane of stele cells shows salt tolerance by selectively transporting Na^+^ from the xylem to the stele regions (Zhang et al., 2012; Chen et al., 2015; Zhang et al., 2019). Interestingly, halophytes living in high-salinity lands exclude sodium and chloride ions from their cells and endure up to 450 mM NaCl. In contrast, glycophytes cannot sustain more than 50 mM NaCl (Munns and Tester, 2008). Halophytes possess a salt-tolerant (exclusion) trait that exploits the HKT family of transporter proteins to prevent the delivery of Na^+^ to the plant shoots, whereas glycophytes do not possess such an efficient functional mechanism (Zhu, 2016; Van Zelm et al., 2020).

HKT belongs to the Trk/Ktr/HKT transporter family (Platten et al., 2006). The HKT transporter family proteins are located in the plasma membrane of xylem parenchyma cells (XPCs) and transport Na^+^ ions from the transpiration stream to the cells, where the ions are sequestered into vacuoles by NHX transporters located in the tonoplast membrane (Horie et al., 2009). Møller et al. (2009) showed that HKT alone reduced the transport of Na^+^ from the roots to shoots by up to 64%. In plants, the HKT protein family has been classified into two subfamilies based on their structure and ion preference (Platten et al., 2006; Horie et al., 2009). Subfamily I preferentially transports Na^+^ in one direction, that is, from the xylem stream to the XPCs. In contrast, subfamily II transports both K^+^ and Na^+^ in a unidirectional direction to the XPCs (Horie et al., 2009). The variation in amino acid residues in the pore loop (P_A_) determines the type, that is, serine in subfamily I and glycine in subfamily II (Horie et al., 2009). These transporters maintain Na^+^ homeostasis in the crop plants. *TaHKT2;1* was first discovered as an HKT transporter in wheat in 1994 (Schachtman and Schroeder, 1994). Plants possess more than one HKT transporter gene in a single genome. However, Arabidopsis possesses only one *AtHKT1;1* transporter gene (Horie et al., 2009). The overexpression of the *HKT* gene in plants improves salt tolerance compared to wild-type plants (Møller et al., 2009; Mian et al., 2011; Xu et al., 2018; Zhang et al., 2019). Natural variations in salinity tolerance have also been demonstrated in HKT transporters in plants. Zhang et al. (2019) explored the natural variation in the *HKT1 (ZmHKT1)* gene in *Zea mays* L. and its salinity tolerance. This study showed that a favorable *HKT1* allele imparts greater salinity tolerance. In addition, the *Na^+^ Content 3* (*ZmNC3*) gene exhibits natural allelic variation and function. Moreover, they identified two haplotypes, HapA with adenine- and HapC with cytosine-associated high and low salt tolerance, respectively. Furthermore, variations associated with gene function were demonstrated in transgenic plants carrying the HapA alleles (Zhang et al., 2019).

*Brassica* is one of the most economically important crops worldwide, contributing to approximately 12% of vegetable oil production globally (Mcalvay et al., 2021). *B. rapa* belongs to the A genome of the *Brassica* genus, and its diploid genome was first sequenced in 2011 (Wang et al., 2011). *B. rapa* is a natural glycophyte (Yepes et al., 2018). With increasing salinity stress worldwide owing to natural calamities and human intervention, developing salt-tolerant crops is important. *B. rapa* is salt-sensitive compared to *B. oleracea*, as *B. rapa*, has a higher Na^+^/K^+^ ratio in leaves than in roots (Pavlović et al., 2019; Hong et al., 2020). Similarly, *B. rapa* had a higher Na^+^/K^+^ ratio in the shoot than *B. oleracea* under salt stress (Pavlović et al., 2019). Overexpression of salt-responsive genes such as RING-H2 finger (*BrATL30*) and zinc-finger homeodomain protein 10 (*BrZHD10*) from *B. rapa* in *B. juncea* has exhibited better salt tolerance (Hong et al., 2020). Under salt stress, *B. napus* showed reduced shoot length and leaf area, which is attributed to salt sensitivity (Hussain Wani et al., 2013). Similarly, *B. juncea* exhibited decreased plant growth and photosynthetic parameters (Hayat et al., 2012).

*B. sinuspersici* is a single-cell C_4_ plant with non-Kranz photosynthetic anatomy (Akhani et al., 2005; Offermann et al., 2015; Koteyeva et al., 2016; Soundararajan et al., 2019). *B. sinuspersici* possesses dimorphic chloroplasts in a single cell, namely central and peripheral chloroplasts, unlike other C_4_ plants (Offermann et al., 2015; Koteyeva et al., 2016; Mai et al., 2019; Han et al., 2023; Won et al., 2023). *B. sinuspersici* is a salt-tolerant halophyte belonging to the family Amaranthaceae *sensu lato* (Kapralov et al., 2012; Han et al., 2023) and possesses salt glands in its leaves. It commonly grows in the saline areas surrounding the Persian Gulf (Akhani et al., 2005; Park et al., 2009; Uzilday et al., 2023). As halophytes complete their life cycle in saline environments, they could be a source of salt-tolerant genes for developing transgenic plants. Moreover, halophytes possess an efficient Na^+^ compartmentalization mechanism. A few studies have been conducted on the salt response of *B. sinuspersici* and have demonstrated salt tolerance (Park et al., 2009; Uzilday et al., 2023). However, no studies have investigated the role of *HKT1* from *B. sinuspersici* in developing salt-tolerant crops.

This study utilized information from an ongoing *B. sinuspersici* genome annotation to identify and characterize *HKT* genes. We studied the *HKTs* of *B. sinuspersici* and their relationships with *HKTs* from other plant lineages. In addition, this study suggests the number of paralogs and their expression patterns in leaves. Then, overexpression of *BsHKT1;2* in the *B. rapa* crop, a glycophyte, with the cauliflower mosaic virus 35S promoter and its characterization was performed. We found that transgenic plants expressing 35S::*BsHKT1;2* showed significantly increased salt tolerance compared to non-transgenic plants. This study provides insights into the *HKT* gene from *B. sinuspersici* and its role in salt tolerance.

## 2 Materials and methods

### 2.1 Plant growth and conditions

Seeds of *B. sinuspersici* (BioProject Accession no. PRJNA273351) obtained from Prof. Sascha Offermann (Institute for Botany, Leibniz University Hannover, 136 Germany), mature *B. rapa* and *A. thaliana* (Col-0), were used in the experiments. All seeds were surface-sterilized using 2% (v/v) sodium hypochlorite for 15 min in a shaker, followed by 20 washes with sterile water. Seeds were sown and grown in Murashige and Skoog (MS) medium with 1% sucrose and 0.8% agar or in a soil mixture under a 16-hour/8-hour photoperiod at 25 °C and 70% humidity. *A. thaliana* seeds were germinated and grown in MS for up to two weeks, and then the plants were used for the salt experiments.

### 2.2 Identification of HKT1

To identify HKT gene families in the *B. sinuspersici* genome, the hidden Markov model (HMM) file of the cation transport protein TrkH (PF02386; https://www.ebi.ac.uk/interpro/entry/pfam/PF02386/) was employed and searched against whole-genome peptides and the transcriptome of *B. sinuspersici* (genome annotation is underway; *BS790c1g1i2* in the transcriptome) (Han et al., 2023). Repeated and truncated proteins were removed. Furthermore, the results were validated by BLAST with well-characterized AtHKT1 (NP567354.1; AT4G10310) proteins from *A. thaliana* against *B. sinuspersici* (**Supplementary Table 1**).

### 2.3 Phylogenetic tree construction and subcellular localization

A phylogenetic tree was constructed for HKT proteins from various plants (**Supplementary Table 2**), *A. thaliana*, and *B. sinuspersici*, using MEGA 11 (Tamura et al., 2021) with ClustalW using the maximum likelihood statistics of 1,000 bootstrap replicates, partial deletion (95), and nearest-neighbor-interchange (NNI) settings. Published HKT proteins belonging to subfamilies 1 and 2 were obtained from the literature and used in the analysis. Furthermore, the P_A_, P_B_, P_C_, and P_D_ domains were examined for subfamily-specific amino acid residues, such as serine (Ser) or glycine (Gly). The DeepLoc-2.0 online tool (https://services.healthtech.dtu.dk/services/DeepLoc-2.0/) was used to predict the subcellular localization of BsHKT proteins (Almagro Armenteros et al., 2017) (**Supplementary Table 3**). For the phylogenetic tree analysis gene, coding sequence, and amino acid sequences in FASTA format were used as inputs (**Supplementary Files. 1-4**).

### 2.4 Gene structural arrangement and motif discovery

To determine the gene structural organization, the genomic position of the HKT1 genes in *B. sinuspersici* was determined using tBLASTn. Contigs encoding HKT1 were retrieved using the SAM tools. The region encoding HKT1 was identified using the FGENESH+HMM profile and extracted using BEDtools. The intron-exon arrangement was mapped using the Gene Structure Display Server 2.0 (GSDS 2.0) online tool (http://gsds.gao-lab.org/index.php) (Hu et al., 2015). Gene and coding sequences (CDS) were used as input into the GSDS 2.0 (**Supplementary Files. 1 and 2**). Conserved HKT1 motifs were identified using the Multiple Em for Motif Elicitation (MEME) Suite web server (https://meme-suite.org/meme/tools/meme) (Bailey et al., 2009) with the zero or one occurrence (ZOOPS) mode, 15 maximum motifs, and 6–200 motif width. Protein sequences were used as inputs (**Supplementary File 3**). Along with BsHKT1;1, BsHKT1;2, and BsHKT1;3, the HKT1 orthologs available in the literature were included in the analysis for comparison (**Supplementary File 4**).

### 2.5 Salt stress imposition

Half-MS medium with 1% sucrose, 0.8% agar, and different salt concentrations (0, 100, 200, and 300 mM NaCl) was prepared. *B. sinuspersici* plants were grown on half-MS media with different salt concentrations in a magenta box for three weeks. The plants were exposed to salt stress on the day of germination. Observations and leaf samples were collected two and three weeks after stress imposition (WAI). Two-week-old *A. thaliana* Col-0 seedlings were also exposed to salt stress. Then, leaf samples were collected one and two days after stress imposition (DAI), snap-frozen in liquid N_2,_ and stored at –80 °C for RNA isolation. For transgenic plants, transgenic seeds were grown on MS media plates for one week. Then, plants were transferred to liquid MS media with different salt concentrations. Wild-type and transgenic plants were subjected to salt stress for seven days. The observations and sample collection were performed at 7 DAI.

### 2.6 Morpho-physiological parameters

Plants subjected to salt stress treatments were observed for variations in root length and foliar canopy growth. At 2 and 3 WAI, plant images were captured using a Canon EOS M50 Mark II (Canon Europe, Amstelveen, the Netherlands), and root length was measured for both treated and untreated plants. Canopy size was also measured. ImageJ software (https://imagej.nih.gov/ij/download/) was used to measure the canopy size.

### 2.7 Gene expression analysis

Total RNA was isolated using the cetyltrimethylammonium bromide (CTAB) method, precipitated in LiCl, and washed with ethanol (Soundararajan et al., 2019). Isolated RNA was dissolved in 30 µL of diethylpyrocarbonate (DEPC)-treated water and stored at –70°C for further analysis. 1 µg of RNA was used for cDNA synthesis (amfiRivert cDNA Synthesis Master Mix; GenDEOPT, USA). Five-fold diluted cDNA was used for real-time quantitative polymerase chain reaction (qPCR) analysis (iQTM SYBR Green^®^ Supermix, Biorad, USA) using the primers mentioned (**Supplementary Table 4**). A two-step SYBR method with 58°C amplification was used with the following conditions in a thermocycler (CFX96 TouchTM Real-Time PCR Detection System; Bio-Rad, USA). Denaturation at 95°C for 3 min, 40 cycles at 95°C for 15 s, 58°C for 30 s, and a melting curve at 95°C for 10 s, 65°C for 5 s, and 60°C for 50 s. Glyceraldehyde-3-phosphate dehydrogenase (GAPDH) was used as an internal control for relative expression analysis. All samples were analyzed using three biological replicates. The relative expression was calculated using the 2^-ΔΔCt^ method.

### 2.8 Construction of the overexpression vector, Brassica transformation, and development of stable lines

A full-length gene of *BsHKT1;2* was amplified from the *B. sinuspersici* genome and cloned into the pCAMBIA1390 vector with a 35S promoter derived from the cauliflower mosaic virus (CaMV35S) using the conventional cloning method in a custom cloning service (http://www.bionicsro.co.kr/). *pCAMBIA1390_35SP_BsHKT1;2* was transformed into Brassica using *Agrobacterium tumefaciens* GV3101. Positive transgenic plants were screened using hygromycin in MS media, and the PCR confirmed the presence of hygromycin phosphotransferase (*hptII*). T1-generation seeds were backcrossed to T3 generations to produce homozygous plants for *BsHKT1;2*.

### 2.9 Flanking DNA sequencing and analysis

Genomic DNA from transgenic and non-transgenic plants was isolated and digested with HaeIII. The digested products were ligated using an adaptor. PCR amplification was performed using primers specific to the T-DNA and adaptor sequences. PCR products were isolated and sequenced using T-DNA-specific primers. The analysis was performed using the flanking sequence tag validator online tool (http://bioinfo.mju.ac.kr/fstval/) (Kim et al., 2012).

### 2.10 Statistical analysis

Statistical analysis was performed using SPSS v16.0 software (https://www.ibm.com/spss) and GraphPad Prism (v5 and 9; https://www.graphpad.com/). One-way or two-way analysis of variance (ANOVA) was used to analyze the significance of differences between wild-type and transgenic lines and treatments. In the two-way ANOVA, a Bonferroni *post-hoc* test was performed to compare the replicate means of non-transgenic plants.

## 3 Results

### 3.1 BsHKTs belong to the HKT subfamily I

**Figure 1:**
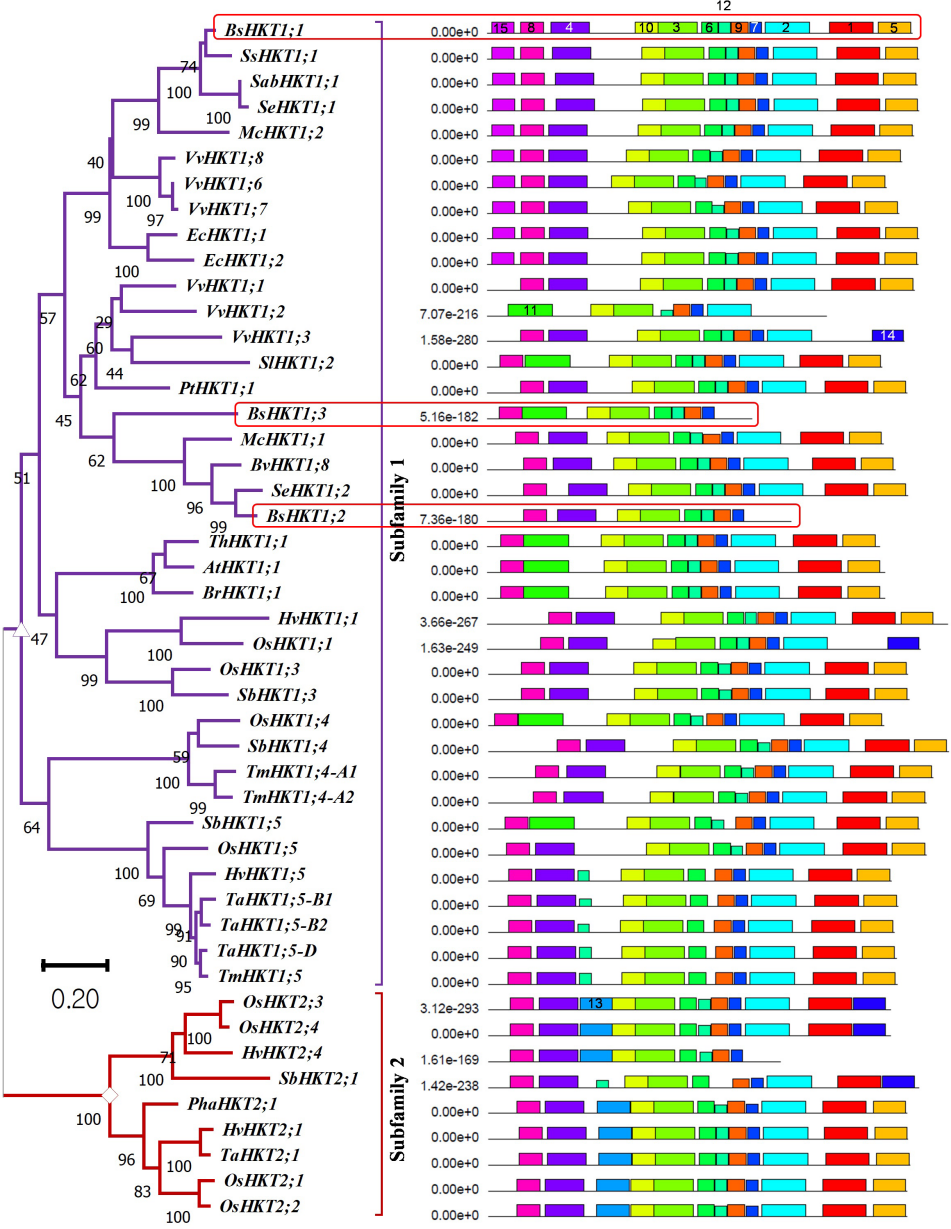
Evolutionary relationships, nomenclature, and motif locations in *BsHKTs*. The unrooted maximum likelihood tree shows an evolutionary relationship with HKTs from higher plants. The name was given based on their similarity to existing HKT proteins. The tree was developed from the data obtained from Platten et al. (2006) and the literature with the addition of HKT protein sequences from *B. sinuspersici*. Evolutionary analyses were conducted in MEGA11 with 1000 bootstrap replicates, and the percentage of replicate trees is shown next to the branches. The tree is drawn to scale, with branch lengths in the same units as those of the evolutionary distances used to infer the phylogenetic tree. Light red square boxes indicate HKTs in *B. sinuspersici*. Scale bar = 0.2 substitutions per site. Species expansion, protein length, and ID details are provided in the **Supplementary Tables 1, 2**. Image (right side) shows the locations of conserved motifs (15) in AtHKT1;1, BsHKT1;1, BsHKT1;2, BsHKT1;3 and other proteins recognized by the MEME motif analysis. Different colors and lengths represent motifs and their lengths, respectively. Amino acid details of identified motifs labeled with numbers and represented by individual colors are provided in **Supplementary Figure 1B**.

Genome-wide identification and a literature survey showed that *A. thaliana* has one copy of *AtHKT1*, which was later renamed *AtHKT1;1* following the subfamily classification (Uozumi et al., 2000). In contrast, three copies of *HKT* were identified in the *B. sinuspersici* genome (**Table 1**). Basic information regarding the three copies of the gene is provided (**Supplementary Table 1**). For phylogenetic analysis, we used protein sequences designated as subfamily I (HKT1) and subfamily II (HKT2) from the literature (**Supplementary Tables 1, 2**). The analysis showed that all three *HKT* genes, *BsHKT1a*, *BsHKT1b*, and *BsHKT1c*, were classified under HKT subfamily I (**Figure 1**). Multiple sequence alignment (MSA) analysis showed that AtHKT1;1 possessed four predominant pore domains. Pore domain A contained a Ser residue at a conserved position. Further analysis at the amino acid sequence level showed that all three BsHKT proteins possessed Ser-Gly-Gly-Gly residues in their P_A_, P_B_, P_C_, and P_D_ domains, respectively. The Ser residues in the P_A_ domain further confirmed that BsHKT1a, BsHKT1b, and BsHKT1c belong to the HKT subfamily I (**Supplementary Figure 1A**). Genomic information on *HKT1* genes is presented in **Table 1**. Based on homology and topology, we assigned the following names to the identified orthologs: *BsHKT1;1*, *BsHKT1;2*, and *BsHKT1;3*.

**Table 1:** Genomic information of *HKT1* gene families in *A. thaliana* and *B. sinuspersici*.

Name	Species	Gene ID	Genome location	Genomic position	Gene length (bp)	CDS length (bp)	Exon count	Protein length (amino acids)
HKT1 family	*A. thaliana*	*AtHKT1:1 (NP_567354.1)*	Chr 4	6391854-6395922	4,069	1,521	3	506
	*B. sinuspersici*	*BsHKT1;1 (Bsv0100-00034511-RA)*	004462F	225257-232481	7,225	1,623	3	541
	*B. sinuspersici*	*BsHKT1;2 (Bsv0100-00016847-RA)*	001276F	370310- 364973	5,338	1,161	2	387
	*B. sinuspersici*	*BsHKT1;3 (Bsv0100-00016849-RA)*	001276F	438683- 440283	1,601	1,011	1	337

HKT, high-affinity K^+^ transporter. Gene sequence, coding sequences, and amino acid sequences are provided in the **Supplementary Files. 1-3**, respectively.

### 3.2 BsHKTs possess domains similar to HKTs belonging to halophytes

MEME is a motif-based sequence analysis tool used to identify conserved motifs (Bailey et al., 2009). The MEME analysis revealed that all three orthologous copies of BsHKT1s from *B. sinuspersici* shared conserved motifs with AtHKT1;1 (**Figure 1**; **Supplementary Figure 1B**). The MEME analysis, with a total of 15 motifs used as input parameters, showed that BsHKT1;1 possessed 12 conserved motifs, except for motifs 11, 13, and 14. In contrast, AtHKT1;1 and BrHKT1;1 had 11 motifs that did not include the motifs 4 and 15, and BsHKT1;2 and BsHKT1;3 possessed eight motifs, lacking the motifs 1, 2, and 5, compared to BsHKT1;1. Moreover, MEME analysis showed the following amino acid sequence variations: HKT subfamily I possessed 14 motifs, except for motif 13, whereas HKT subfamily II contained 13 conserved motifs, except for motifs 11 and 15. BsHKT1;1 possessed several motifs similar to those of EcHKT1;1, EcHKT1;2, McHKT1;2, SabHKT1;1, SeHKT1;1, SsHKT1;1, VvHKT1;6, VvHKT1;7, and VvHKT1;8. In contrast, BsHKT1;2, BsHKT1;3, and VvHKT1;3 possessed fewer motifs. The least number of motifs were found in VvHKT1;2 (N=7) and HvHKT2;4 (N=9) in the HKT1 and HKT2 families, respectively. All HKTs contained motifs 3, 7, 9, and 10 (**Figure 1**; **Supplementary Figure 1B**). Subcellular localization prediction analysis showed that BsHKT1;1, BsHKT1;2, and BsHKT1;2 were localized in the plasma membrane, similar to AtHKT1;1 (Almagro Armenteros et al., 2017) (**Supplementary Table 3**).

### 3.3 *BsHKTs* contain variable numbers of introns and exons

The GSDS 2.0 online tool (Hu et al., 2015) was used to investigate the arrangement of introns and exons in the *BsHKT* genes. The analysis showed that *BsHKT1;1* had three exons and two introns, similar to *AtHKT1;1* structure (**Supplementary Figure 1C**). However, the length of *BsHKT1;1* was almost double that of *AtHKT1;1*. Notably, the other two paralogs, *BsHKT1;2* and *BsHKT1;3*, contained only two and one exon, respectively. Moreover, *BsHKT1;2* has only one intron, whereas *BsHKT1;3* has no introns. Furthermore, the length of *BsHKT1;2* was similar to *AtHKT1;1*, whereas *BsHKT1;3* was shorter than *AtHKT1;1* (**Supplementary Figure 1C**).

### 3.4 Bienertia grows better in salt medium with higher *BsHKT1;2* expression

**Figure 2:**
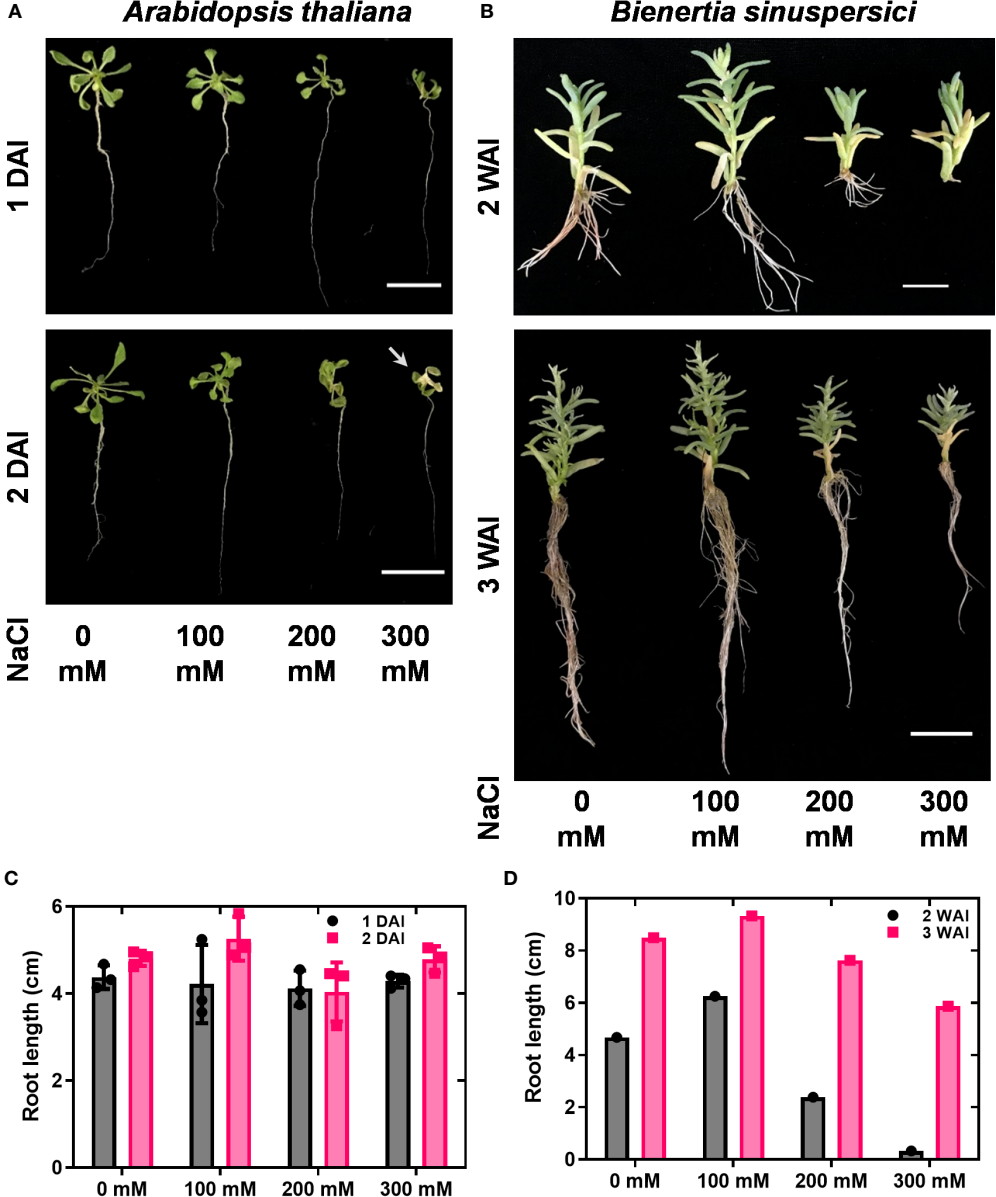
Shoot and root phenotypes under salt stress. *A. thaliana* and *B. sinuspersici* were subjected to salt stress (0, 100, 200, and 300 mM). NaCl was used as a salt component. *A. thaliana* and *B. sinuspersici* plants were transferred to ½ MS media containing different salt concentrations two weeks after sowing and subjected to salt stress. *A. thaliana* plants were observed for phenotype changes 1 and 2 DAI, whereas *B. sinuspersici* plants were observed for phenotype changes 2 and 3 WAI. **(A)** Images show the *A. thaliana* phenotype at 1 DAI and 2 DAI under different salt concentrations. **(B)** Images show the *B. sinuspersici* phenotype at 2 and 3 WAI. **(C, D)** graphs show the root lengths of *A. thaliana* and *B. sinuspersici* under different salt concentrations, respectively. Scale bar: 2 cm. Error bar= standard error of the mean. N=4 for *A. thaliana* and N=1 for *B. sinuspersici*. Note: the white arrow points out the foliar yellowing caused by salt stress. DAI, days after stress imposition; WAI, weeks after stress imposition.

To investigate the expression patterns of *HKT* genes under salt stress conditions, wild-type plants were treated with 0, 100, 200, and 300 mM NaCl. As the growth of *A. thaliana* was severely affected over two days under *in vitro* conditions, samples were collected one and two DAI. In *A. thaliana*, reductions in growth, leaf scorching symptoms, and root inhibition have been observed under salt stress. Overall, the salt treatment diminished the growth of *A. thaliana* (**Figure 2A**). For *B. sinuspersici*, salt treatment was conducted for two and three weeks.

**Figure 3:**
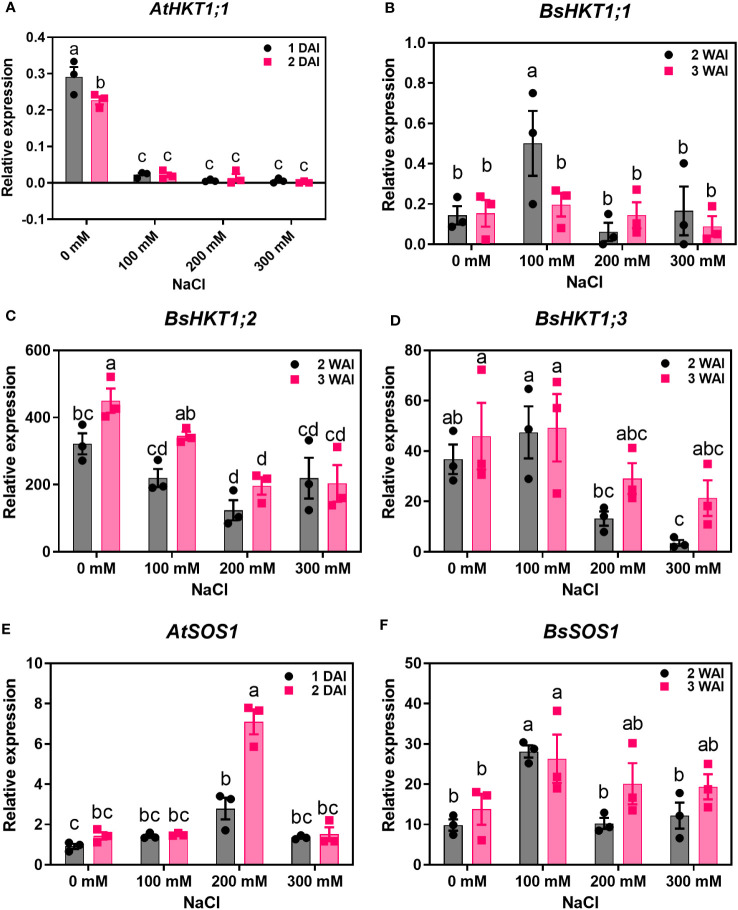
Quantitative real-time PCR results of *HKTs* and *SOS1* genes under salt stress. *A. thaliana* and *B. sinuspersici* were subjected to salt stress (0, 100, 200, and 300 mM), and RNA was isolated from shoots 1 and 2 DAI for *A. thaliana*, whereas for *B. sinuspersici* plants, at 2 and 3 WAI. Graphs show the relative expression levels of **(A)**, *AtHKT1;1*; **(B)**, *BsHKT1;1*; **(C)**, *BsHKT1;2*; **(D)**, *BsHKT1;3*; **(E)**, *AtSOS1* and **(F)**, *BsSOS1* to the internal control *BrGAPDH* under 0 mM NaCl. Error bar= standard error of the mean. N=3. The letters above the column indicate the significant difference among the treatments. DAI, days after stress imposition; WAI, weeks after stress imposition.

Improved growth of *B. sinuspersici* was observed in the 100 mM NaCl treatment compared to the control. In contrast, 200 and 300 mM NaCl reduced growth and decreased root length in plants compared to the control (**Figures 2B–D**). In this study, the expression levels of three orthologous copies, *BsHKT1;1, BsHKT1;2, and BsHKT1;3* from the HKT1 family and *SOS1* from the NHX family, under salt stress conditions were determined. Decreased expression of *AtHKT1;1* was observed at all salt concentrations. The *BsHKT1;1*, *BsHKT1;2*, and *BsHKT1;3* expression levels were strongly increased in *B. sinuspersici* treated with 100 mM NaCl. Higher expression of *BsHKT1;2* was observed at moderate and high NaCl concentrations (0, 100, and 200 mM) at 3 WAI than at 2 WAI, except at 300 mM NaCl. In contrast, a higher expression of *BsHKT1;3* was observed at exceptionally high NaCl concentrations (200 and 300 mM) at 3 WAI than at 2 WAI. Importantly, while increased expression of *BsSOS1* in *B. sinuspersici* was observed at 200 and 300 mM NaCl, *AtSOS1* in *A. thaliana* did increase its expression level at 300 mM NaCl at 2 DAI. In addition, extremely high expression of *AtSOS1* was detected in the presence of 200 mM NaCl at 2 DAI. Interestingly, *BsHKT1;3* and *BsSOS1* showed similar gene expression patterns under salt stress. The maximum level of *BsSOS1* expression was observed at 100 mM NaCl (**Figure 3**).

### 3.5 Homozygous transgenic *B. rapa* plants overexpress *BsHKT1;2* under the 35S promoter

**Figure 4:**
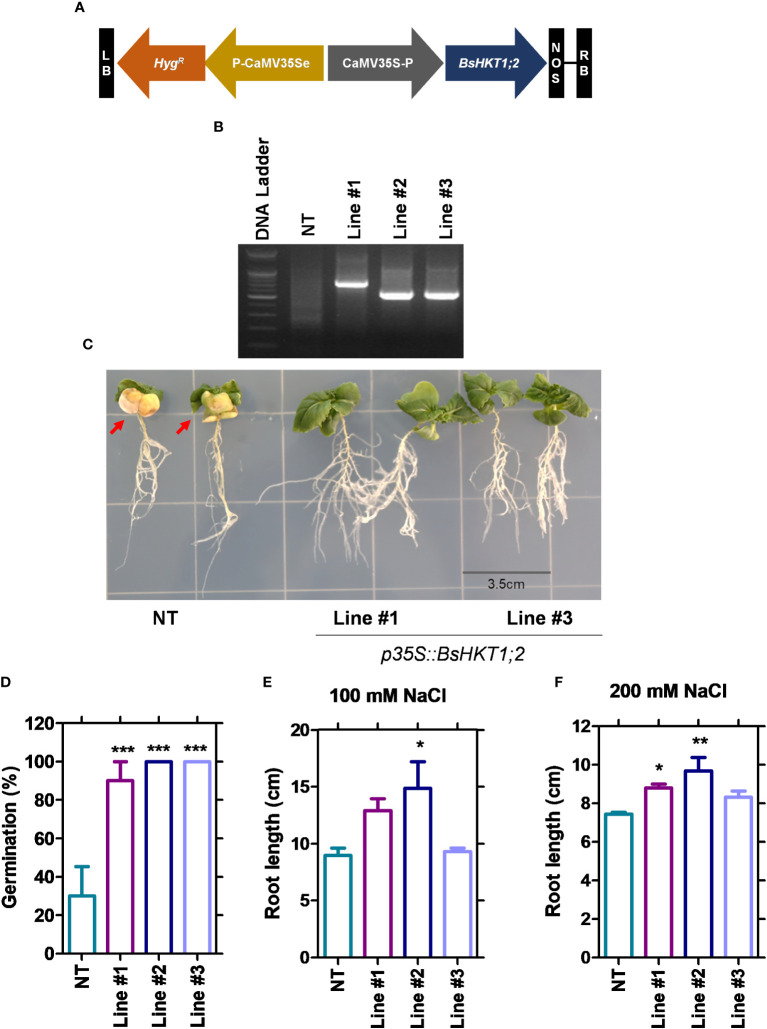
Morpho-physiological responses of transgenic plants under salt stress. NT and transgenic plants were subjected to salt stress (150 mM), and observation was carried out on 7 DAI. **(A)** The diagram shows the *BsHKT1;2* gene construct in vector pCAMBIA1390. **(B)** The traditional cloning result is confirmed by restriction enzymes. **(C)** The image panel shows the foliar and root phenotypes of NT and transgenic plants subjected to 100 and 200 mM salt stress, respectively. **(D)** The graph shows the percentage of germination of the NT and transgenic plants. **(E, F)** The graphs show the root length of the NT and transgenic plants under 100 and 200 mM NaCl. The error bar indicates the standard error of the mean. A one-way ANOVA was performed, followed by Tukey’s multiple comparison test. Asterisks indicate the significance (* p < 0.05; **p < 0.01; ***p < 0.001). N=3. The experiment was at least repeated twice. Note: Line #1, 4-4; Line #2, 5-8; Line #3, 5-9; NT, non-transgenic plant. Red arrows indicate the yellowing foliar symptom in NT plants. DAI, days after stress imposition.

Since *BsHKT1;2* showed increased expression levels (**Figure 3**; **Supplementary Figure 2A**), to further investigate its efficiency in salt tolerance, the full-length gene was amplified from cDNA and cloned into pCAMBIA1390 using the cauliflower mosaic virus 35S promoter (CaMV35S) (**Figures 4A, B**). The primer details are shown in **Supplementary Table 4**. *B. rapa* was transformed with *35S::BsHKT1;2* using *A. tumefaciens*. Transformed plants were initially screened under hygromycin antibiotics in MS medium for four rounds of plant transfer, during which the chimeric plants were removed. PCR amplification and plant mortality in salt media confirmed that transgenic *B. rapa* contained the *35S::BsHKT1;2* segments (**Figures 4B, D**). Then, the expression levels were measured. Positive plants were confirmed by PCR, and T_1_-positive plants were backcrossed for up to two generations (**Supplementary Figure 3**). From the segregation analysis, stable lines (T_2_ generation) were selected and used in salt stress experiments.

### 3.6 *BsHKT1;2* is inserted into the 5’ upstream and intron regions

Flanking DNA sequencing analysis was performed (**Supplementary Figure 4**) and showed that *BsHKT1;2* genes were inserted at the 1 kb 5’ upstream of the DnaJ domain in line #1 (4-4) and at the intron regions of a protein with unknown function in line #2 (5-8) and #3 (5-9) (**Table 2**). Irrespective of the insertion location, transgenic plants were healthy and did not show a slight change in plant morphology, similar to non-transgenic plants.

**Table 2:** Genomic details on *BsHKT1;2* insertion locations in the *B. rapa* genome based on flanking sequence tag validator (FSTVAL) analysis.

S. No	Query Name	Chr_start	Chr_end	Type	Gene_id	Description
1	*Br_BsHKT1;2* gene_line #1	14299150	14298961	5’Upstream-1000	*Bra008278* 0.207 Kb	DnaJ domain; Myb-like DNA-binding domain
2	*Br_BsHKT1;2* gene_line #2	2441742	2441815	Intron	*Bra036152*	Protein of unknown function (DUF581)
3	*Br_BsHKT1;2* gene_line #3	2441742	2441815	Intron	*Bra036152*	Protein of unknown function (DUF581)

Line #1, 4-4; Line #2, 5-8; Line#3, 5-9. FSTVAL online tool (Kim et al., 2012) (http://bioinfo.mju.ac.kr/fstval/).

### 3.7 BsHKT1;2 in *B. rapa* imparts salt tolerance under salt stress

Stable transgenic and non-transgenic lines were exposed to 0, 100, or 200 mM salt concentrations (NaCl) in the MS medium (**Supplementary Figure 5**). The growth of the transgenic plants was similar to that of non-transgenic plants under 0 mM salt (non-salt conditions). However, at 100 mM NaCl, the growth of the transgenic plants was greater than that of non-transgenic plants. Transgenic plants had green canopies, relatively larger leaves, longer roots, and grew faster than non-transgenic plants (**Figure 4C**). However, root length decreased in both the non-transgenic and transgenic lines with increasing salt concentrations (100 and 200 mM) (**Figures 4E, F**). The *BsHKT1;2* expression levels in the transgenic plants were quantified, along with *BrNHX1.1*, *BrNHX1.2*, *BrNHX1.3*, *BrNHX2*, *BrNHX3.2*, and *BrNHX7.*

**Figure 5:**
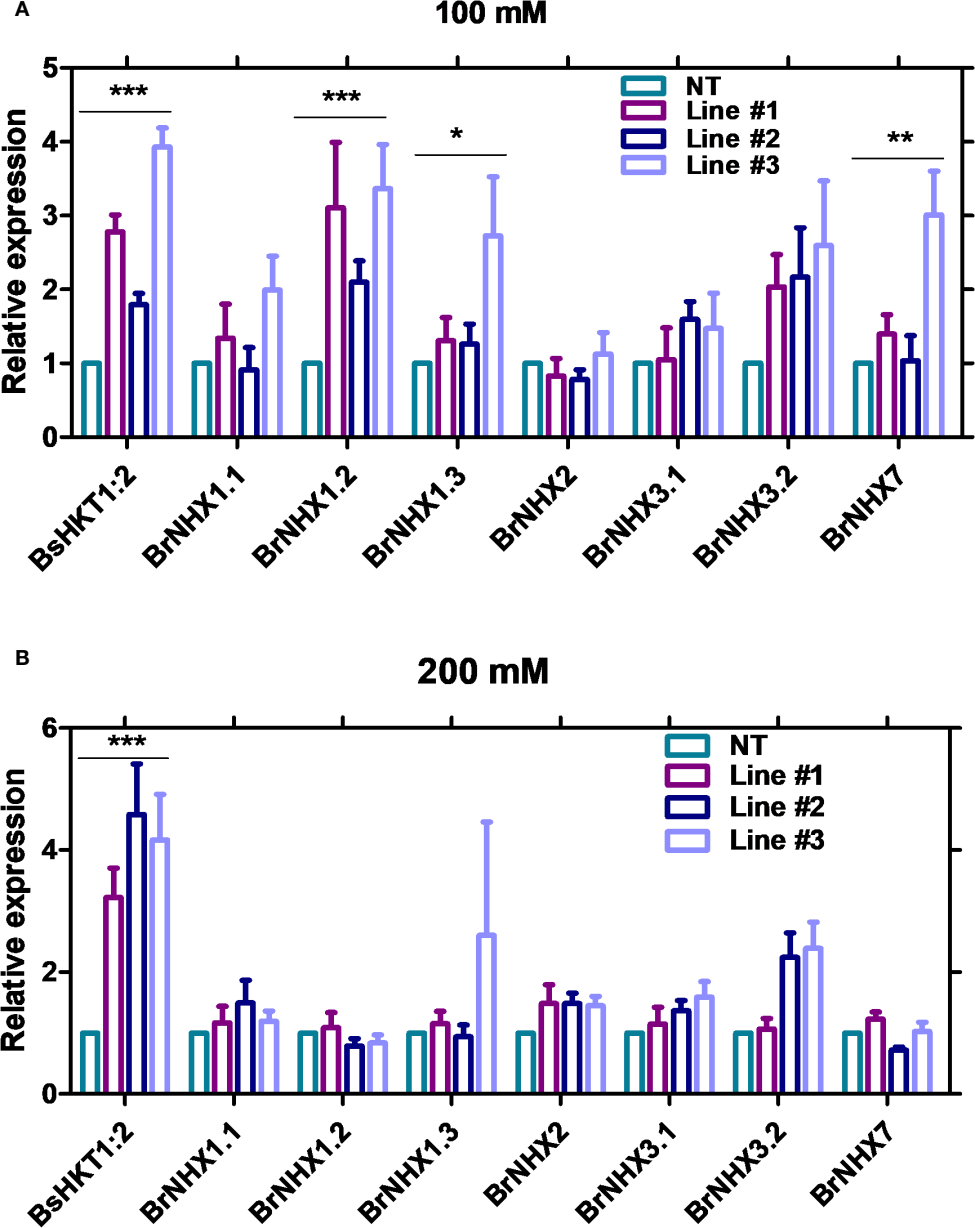
Quantitative real-time PCR results of salt stress-associated genes in transgenic *B. rapa*. Non-transgenic plants (NT) and *35S::BsHKT1;2* transgenic plants (lines#1-3) were subjected to salt stress (0, 100, and 200 mM NaCl). Plant samples were collected 7 DAI, and gene expression was quantified. Graphs show the relative gene expression level of *BsHKT1;2* and salt stress-associated genes under 100 **(A)** and 200 mM salt stress **(B)**. The error bar indicates the standard error of the mean. A two-way ANOVA was performed, followed by a Bonferroni post-test. Asterisks indicate the significance (* p < 0.05; **p < 0.01; ***p < 0.001). N=3. The experiment was at least repeated twice. Note: Line #1, 4-4; Line #2, 5-8; Line#3, 5-9; NT, non-transgenic plant expression levels. DAI, days after stress imposition.

The results showed that the expression level of *BsHKT1;2* was 2–4-fold higher at 100 mM salt stress and up to 5-fold higher under 200 mM salt stress in transgenic plants than in non-transgenic plants. Moreover, the *BrNHX1.2*, *BrNHX1.3*, *BrNHX3.2*, and *BrNHX7* expression levels were higher under 100 mM NaCl, whereas their expression levels were not different at 200 mM NaCl compared to non-transgenic plants (**Figures 5A, B**).

## 4 Discussion

Plants experience salt stress in soils with electrical conductivity of saturated soil paste extract (ECe) of 4 deciSiemens per metre (dS/m). Crop plants, especially glycophytes, exhibit salt stress symptoms in soils with ECe <4 dS/m (Munns and Tester, 2008). Plants possess HKT transporters that decrease the transport of Na^+^ from the root to the shoot. The heterologous expression of *AtHKT1;1* from Arabidopsis in oocytes has been shown to be a Na^+^-selective uniporter (Uozumi et al., 2000). The HKT transporter was explored in the *B. rapa* genome. *BrHKT1;1* (XP_009134189.2) was predicted through automated computational analysis from a genomic sequence (NC_024797.2) (Zhang et al., 2018). However, no further analysis was available. This may be attributed to the glycophyte nature of *B. rapa*. Several studies have strengthened the potential of HKT transporters to decrease and increase Na^+^ content in shoots and roots, respectively (Uozumi et al., 2000; Xu et al., 2018; Wang et al., 2019). Interestingly, *HKT* expression was observed in both root and shoot tissues. However, their functions varied. In the root, it sequesters Na^+^ ions into XPCs, whereas in the shoot, *HKT* transports excess Na^+^ from the leaf parenchymal cells to the phloem stream (Mian et al., 2011), which is attributed to salt tolerance. In this study, we investigated the role of the salt-responsive *BsHKT1:2* in a terrestrial halophyte plant *B. sinuspersici*. Particularly, we studied the structural classification and functional characteristics of the BsHKT transporters. *B. sinuspersici* is an SCC_4_ plant that thrives in salt-affected areas and is commonly found around the Persian Gulf and Oman Sea, in southwestern Pakistan, southern Iran, Kuwait, southern Iraq, Saudi Arabia, Qatar, and the UAE (Akhani et al., 2005; Akhani et al., 2012; Uzilday et al., 2023). As Bienertia is a halophyte (Park et al., 2009), we assumed that it has efficient mechanisms for coping with salt stress. Therefore, we performed genome annotation and identified the *HKT* and salt overly sensitive 1 (*SOS1*) genes in the *Bienertia* genome. Three paralogs of the HKT family were identified: *BsHKT1;1*, *BsHKT1;2*, and *BsHKT1;3* (**Figure 1**, **Table 1**, and **Supplementary Table 1**). Studies have demonstrated that the rice genome contains nine paralogs of *HKT* genes (Garciadeblás et al., 2003; Jabnoune et al., 2009). Of these, five were classified as HKT subfamily II and four as HKT subfamily I (Garciadeblás et al., 2003). Similarly, barley possesses five paralogs of *HKT* (Hazzouri et al., 2018). In addition, variations in the number of paralogs were demonstrated between durum wheat and bread wheat genomes (Huang et al., 2008). Most monocot genomes contain HKT proteins belonging to subfamily II, whereas dicots contain HKT proteins belonging to subfamily I (Platten et al., 2006). Furthermore, monocot genomes contain more than one copy of the HKT protein (Garciadeblás et al., 2003; Platten et al., 2006; Jabnoune et al., 2009). Based on the phylogenetic clusters of Platten et al. (2006), our phylogenetic analysis, which included HKT proteins representing two subfamilies, showed that all three HKTs from *B. sinuspersici* were classified as HKT subfamily I (**Figure 1**; **Supplementary Table 2**). Most HKT from diploid plants contains the Ser residue at pore domain A, which transports Na^+^ from the transpiration stream to XPCs (Horie et al., 2009; Møller et al., 2009; Wang et al., 2020). Particularly, HKT proteins in the family Amaranthaceae *sensu lato* are classified into subfamily I because they have Ser-Gly-Gly-Gly residues in four major pore domains (Flowers and Colmer, 2008; Wang et al., 2020).

Moreover, motif analysis showed that BsHKT1;1 had motifs similar to those of AtHKT1;1 and BrHKT1;1; however, it had one motif replacement and one extra motif compared with AtHKT1;1 (**Figure 1**). However, fewer motifs were identified in BsHKT1;2 and BsHKT1;3. HKT proteins from halophytes possess complete motifs. However, the function of BsHKT1;1 may differ from those of BsHKT1;2 and BsHKT1;3. Interestingly, although most glycophytes contained a large number of motifs, a few HKTs contained a small number of motifs and were barely similar to *Bienertia* and grapevine. A study reported that Criolla grapevine cultivars exhibited greater salt resistance than other cultivars, and *AtNHX1* transgenic grapevines showed enhanced salt tolerance (Venier et al., 2018). Therefore, we assumed that the co-regulation of HKT and NHX may be important for fine-tuning the mechanism of salt resistance. In addition, both OsHKT1;1 and OsHKT2;1 lacked the motif 15. The arrangement and reduction in the number of motifs were attributed to the evolution of the genes. *BsHKT1;1* possesses three exons and two introns (**Supplementary Figure 1C**), similar to *AtHKT1;1* (Platten et al., 2006). However, *BsHKT1;2* and *BsHKT1;3* contained two exons and one exon, respectively. Most HKT genes contain three exons (Uozumi et al., 2000; Platten et al., 2006; Jabnoune et al., 2009). However, HKT genes from *Vitis vinifera* (*VvHKT3a2*) possess four exons (Li et al., 2019). Similarly, *CsHKT3b* from *Cucumis sativus* contains 11 exons. Moreover, variation in the number of introns was observed. For example, *GmHKT3c4* from *Glycine max* contains only one intron (Li et al., 2019). The reason behind the loss of exons and introns may be attributed to convergent evolution.

Salt tolerance is a complex process. Entering Na^+^ in plant root cells triggers an elevation in Ca^2+^ in the cytoplasm. In turn, SOS3 perceives the increased Ca^2+^ levels and activates SOS2, a protein kinase. Consequently, SOS2 translocates to the plasma membrane and phosphorylates SOS1, facilitating the extrusion of Na^+^ into the soil (Ali et al., 2023; Liu et al., 2023). Under salt stress conditions, the differential expression of *BsHKT1;1*, *BsHKT1;2*, *BsHKT1;3*, and *BsSOS1* in *B. sinuspersici* under different concentrations of NaCl showed that the increased expression level of *BsHKTs* slightly improved plant growth at 100 and 200 mM NaCl, whereas decreased expression of *BsHKTs* at 300 mM NaCl arrested plant growth. Overall, increasing salt concentrations affected the *HKT* expression. However, *BsHKT1;2* was highly expressed among the three *BsHKTs*. Probably, the extremely increased expression of *BsHKT1;2* is due to *BsHKT1;2* transports more Na^+^ into the xylem parenchyma. Moreover, increased *BsHKT1;2* expressions (CT value=22.6 ± 0.2 under control conditions) indicates that it may participate in salt stress signaling. Further detailed research on *BsHKT1;2* will support plant salt stress signaling.

**Figure 6:**
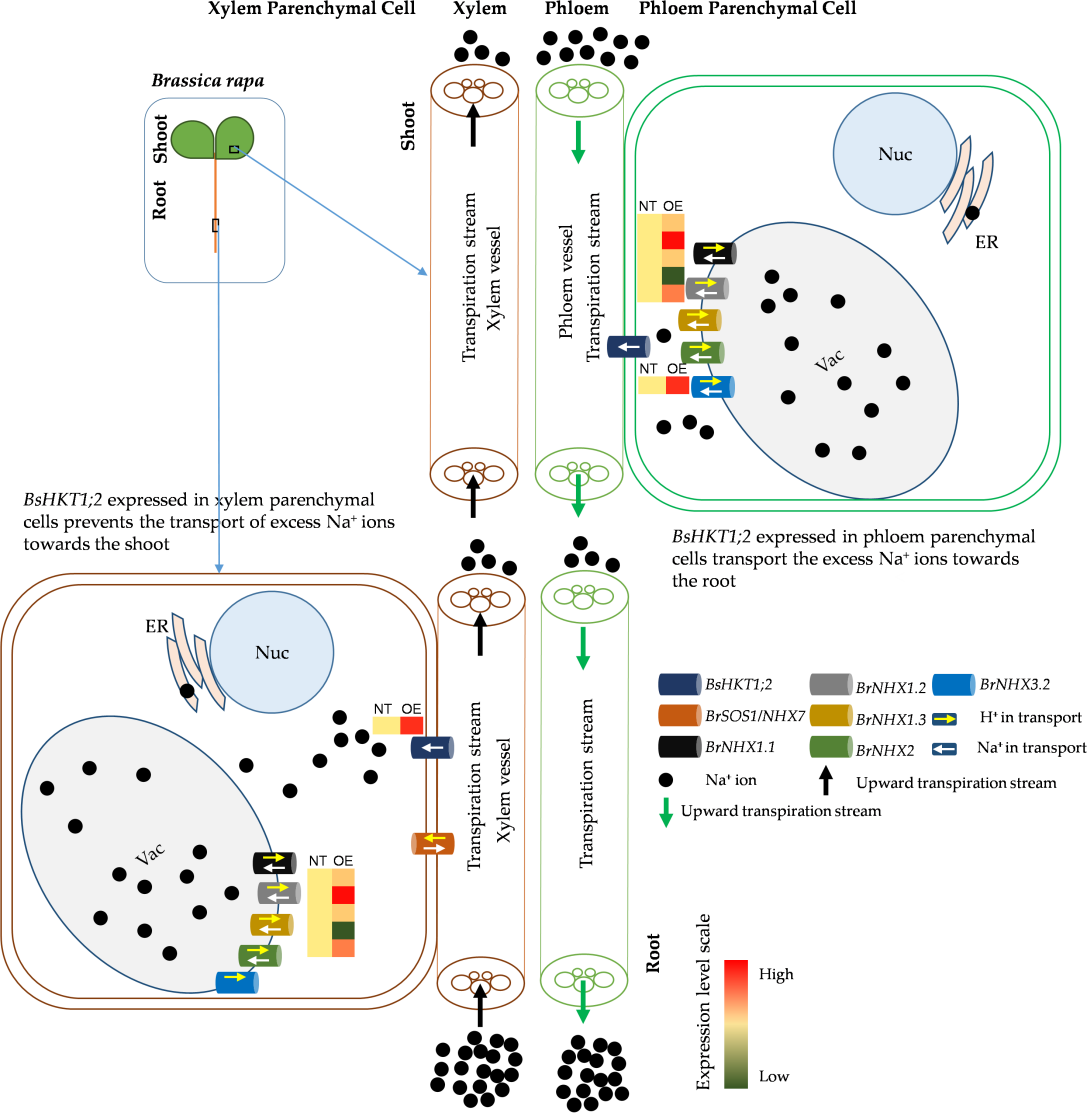
Schematic diagram of improved salt stress tolerance in transgenic *BsHKT1;2* plant. Plants uptake Na^+^ ions through non-selective cation channel transporters located in the root epidermal cells. Then, Na^+^ ions travel toward the shoot in the xylem transpiration stream. HKT transporters present in the xylem parenchymal cell (XPC) membranes transport the Na^+^ ions into the XPC in a unidirectional way. Na^+^/H^+^ exchangers (NHXs) further transport the excess Na^+^ ions into vacuoles bidirectionally with the exchange of H^+^ ions. In this study, we found that increased expression of *BsHKT1;2* in the transgenic *B. rapa* imparted salt tolerance by transporting more Na^+^ into XPC from the transpiration stream. Also, BsHKT1;2 located in phloem parenchymal cells in the foliar transport excess Na^+^ ions into the phloem vessel, which in turn are extruded out by the NHX7 transporter. Vac, vacuole; Nuc, nucleus; ER, endoplasmic reticulum; NT, non-transgenic; OE, overexpressing transgenic lines; Na^+^, sodium ions; H^+^, protons.

Decreased expression of *AtHKT1;1* and *BrHKT1;1* was observed at all salt concentrations, which may be because both *AtHKT1;1* and *BrHKT1;1* is salt sensitive due to the *A. thaliana* and *B. rapa* being a glycophyte (Shi et al., 2003). HKT overexpression improves salinity tolerance in models and crop plants (Shi et al., 2003; Xu et al., 2018; Wang et al., 2019). When transgenic plants were exposed to salt stress, all plants showed higher expression levels than the control plants, indicating that *BsHKT1;2* is functional in *B. rapa* (**Figure 6**). Morpho-physiological parameters, such as biomass, canopy size, and root length, showed that *B. rapa* with *35S::BsHKT1;2* was salt tolerant. In addition, BsHKT1;2 in the cell membrane strengthens the idea that the highly expressed BsHKT1;2 in transgenic plants under salt stress transports more Na^+^ into XPC. In addition, under 200 mM NaCl, the high expression of *BsHKT1;2* and low expression of NHX transporters indicate that *BsHKT1;2* may play a role in transporting Na^+^ into vascular organelles. However, studies have proven that HKT proteins are generally located in the XPC plasma membrane (Uozumi et al., 2000; Horie et al., 2009; Mian et al., 2011). Further studies need to be conducted to understand the interaction between *SOS2* and *BsHKT1;2* under salt stress. Hence, the results of this study imply that *BsHKT1;2* is more efficient in transporting Na^+^ and imparts salt tolerance to *B. sinuspersici*. In addition, when *BsHKT1;2* was overexpressed in *B. rapa*, salt tolerance was increased to maintain its productivity.

## Data availability statement

The datasets presented in this study can be found in online repositories. The names of the repository/repositories and accession number(s) can be found below: https://www.ncbi.nlm.nih.gov/protein/WOK82374.1, https://www.ncbi.nlm.nih.gov/protein/WOK82375.1, https://www.ncbi.nlm.nih.gov/protein/WOK82376.1.

## Author contributions

VI: Data curation, Investigation, Validation, Visualization, Writing – original draft. HP: Investigation, Resources, Validation, Writing – review & editing. SH: Data curation, Investigation, Writing – review & editing. MK: Data curation, Validation, Writing – review & editing. JK: Conceptualization, Funding acquisition, Project administration, Resources, Supervision, Writing – review & editing.
