# Supplementary material for: Genome-wide identification of a novel Na+ transporter from Bienertia sinuspersici and overexpression of BsHKT1;2 improved salt tolerance in Brassica rapa
Source: Front Plant Sci. 2023 Dec 12;14:1302315. doi: 10.3389/fpls.2023.1302315 (PMC10773568; doi:10.3389/fpls.2023.1302315)
Supplement: Supplementary file 1 [file DataSheet_1.zip › Supplementary Figures.pptx]

## Slide 1
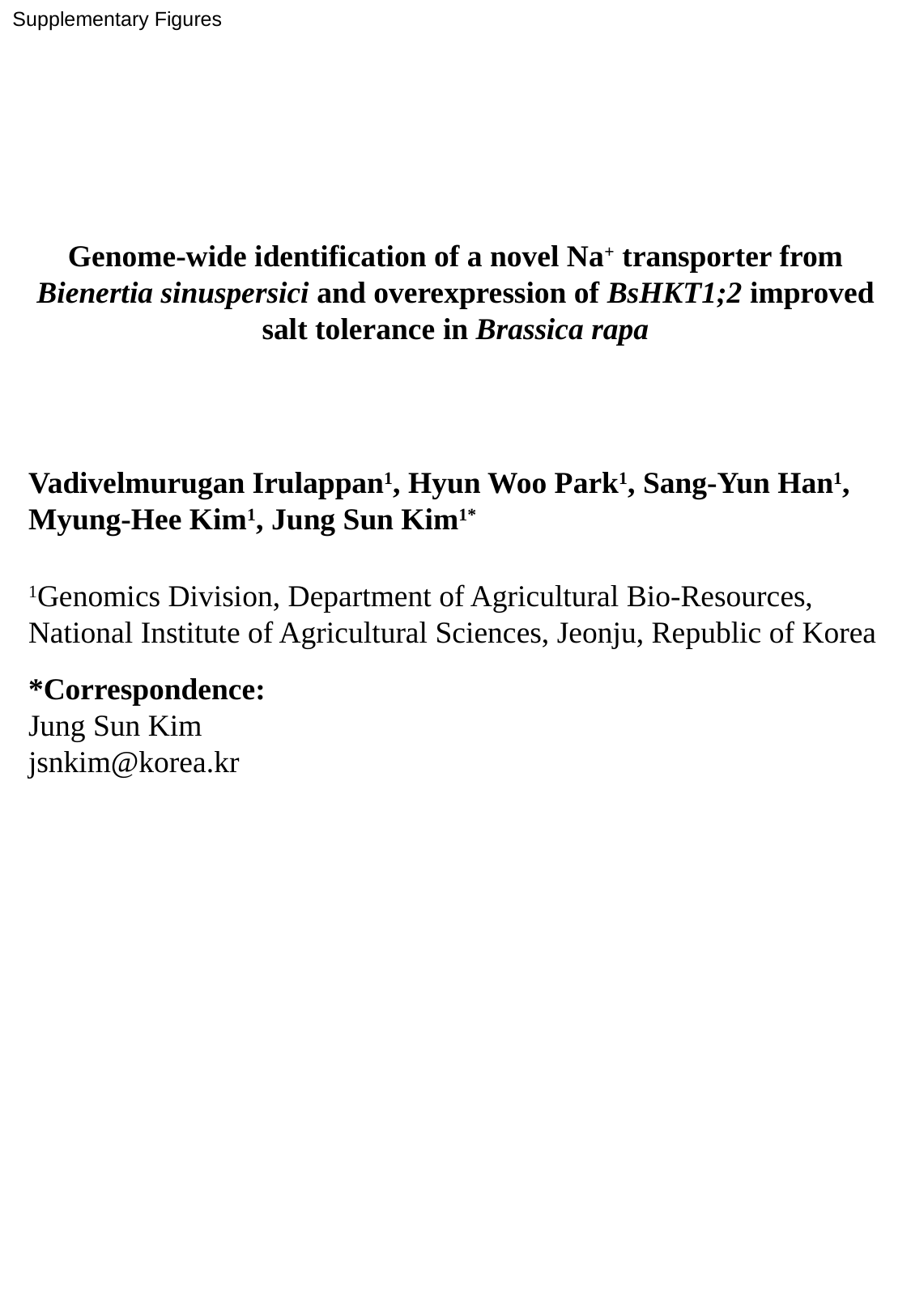

Supplementary Figures
Genome-wide identification of a novel Na+ transporter from Bienertia sinuspersici and overexpression of BsHKT1;2 improved salt tolerance in Brassica rapa
Vadivelmurugan Irulappan1, Hyun Woo Park1, Sang-Yun Han1, Myung-Hee Kim1, Jung Sun Kim1*
1Genomics Division, Department of Agricultural Bio-Resources, National Institute of Agricultural Sciences, Jeonju, Republic of Korea
*Correspondence:
Jung Sun Kim
jsnkim@korea.kr

## Slide 2
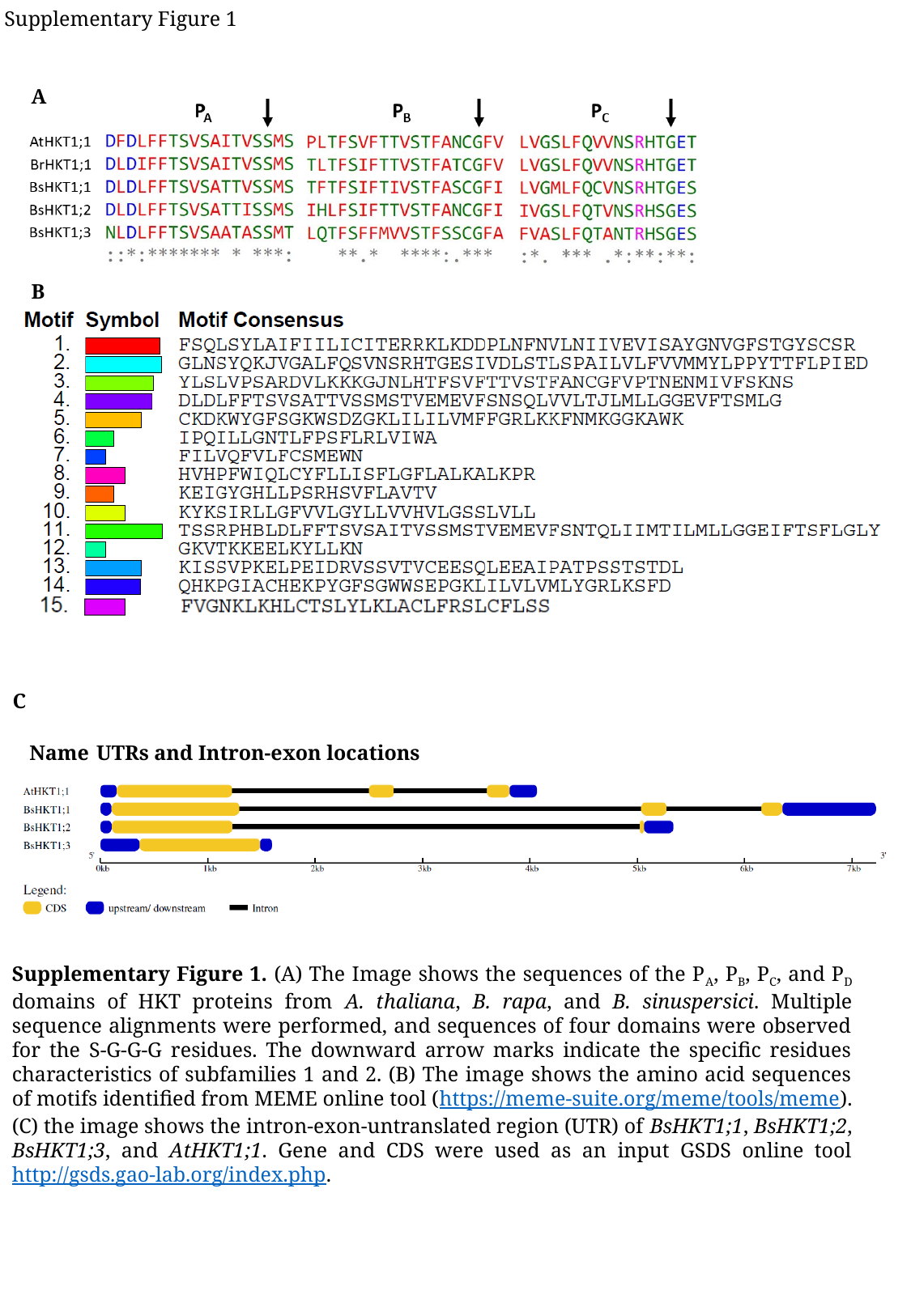

Supplementary Figure 1
A
B
C
Name
UTRs and Intron-exon locations
Supplementary Figure 1. (A) The Image shows the sequences of the PA, PB, PC, and PD domains of HKT proteins from A. thaliana, B. rapa, and B. sinuspersici. Multiple sequence alignments were performed, and sequences of four domains were observed for the S-G-G-G residues. The downward arrow marks indicate the specific residues characteristics of subfamilies 1 and 2. (B) The image shows the amino acid sequences of motifs identified from MEME online tool (https://meme-suite.org/meme/tools/meme). (C) the image shows the intron-exon-untranslated region (UTR) of BsHKT1;1, BsHKT1;2, BsHKT1;3, and AtHKT1;1. Gene and CDS were used as an input GSDS online tool http://gsds.gao-lab.org/index.php.

## Slide 3
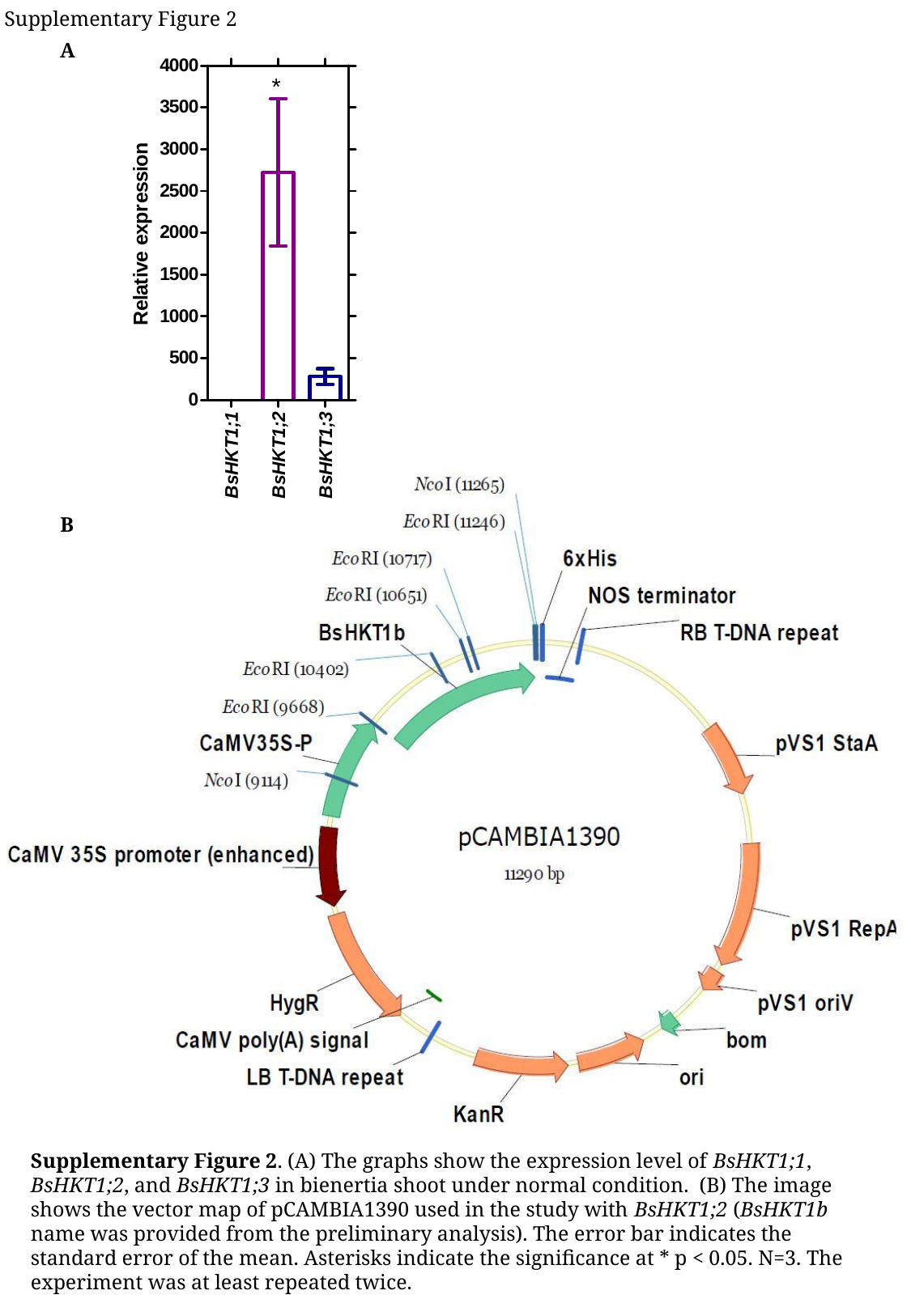

Supplementary Figure 2
A
B
Supplementary Figure 2. (A) The graphs show the expression level of BsHKT1;1, BsHKT1;2, and BsHKT1;3 in bienertia shoot under normal condition. (B) The image shows the vector map of pCAMBIA1390 used in the study with BsHKT1;2 (BsHKT1b name was provided from the preliminary analysis). The error bar indicates the standard error of the mean. Asterisks indicate the significance at * p < 0.05. N=3. The experiment was at least repeated twice.

## Slide 4
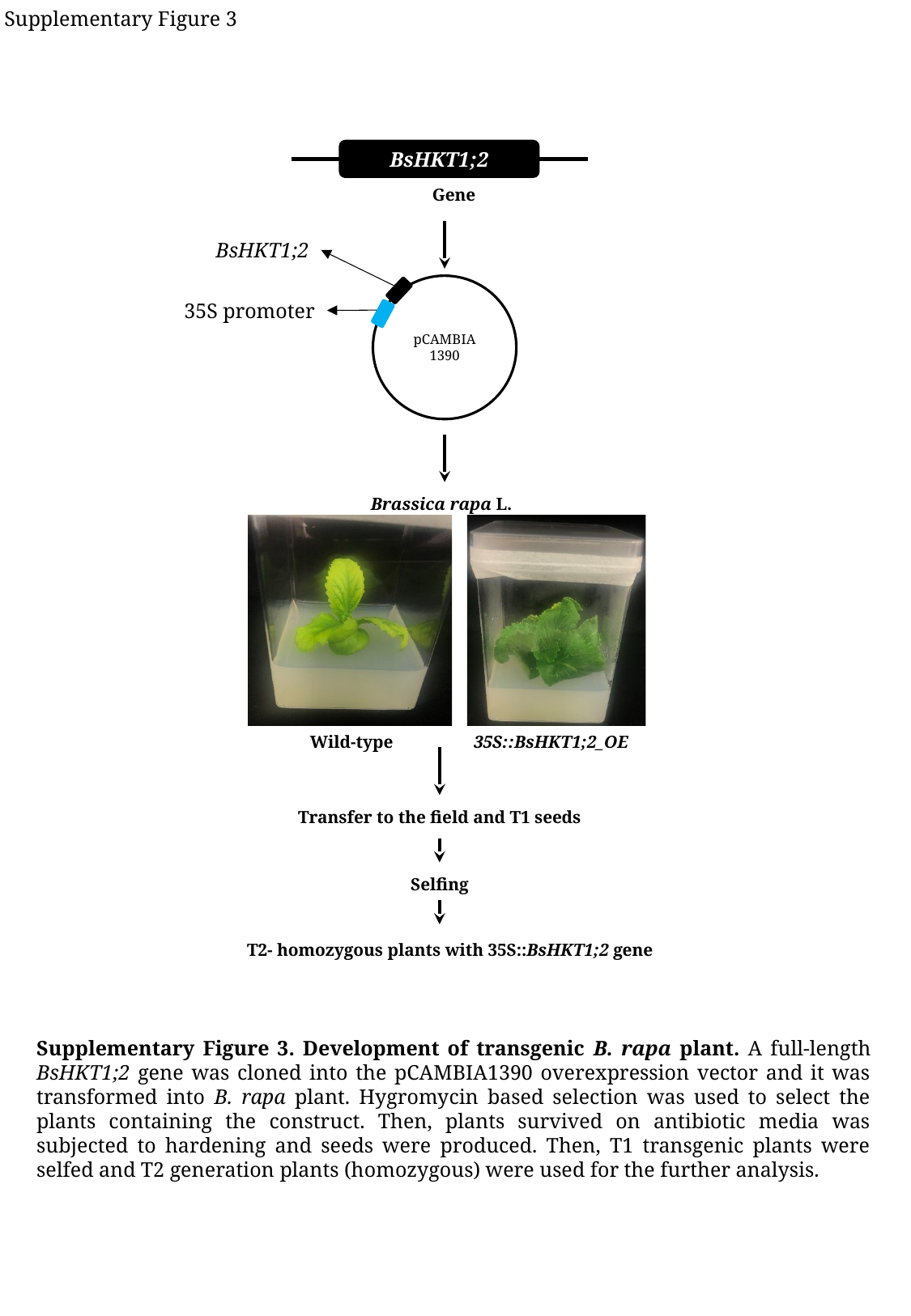

Supplementary Figure 3
BsHKT1;2
Gene
BsHKT1;2
pCAMBIA
1390
35S promoter
Brassica rapa L.
Wild-type
35S::BsHKT1;2_OE
Transfer to the field and T1 seeds
Selfing
T2- homozygous plants with 35S::BsHKT1;2 gene
Supplementary Figure 3. Development of transgenic B. rapa plant. A full-length BsHKT1;2 gene was cloned into the pCAMBIA1390 overexpression vector and it was transformed into B. rapa plant. Hygromycin based selection was used to select the plants containing the construct. Then, plants survived on antibiotic media was subjected to hardening and seeds were produced. Then, T1 transgenic plants were selfed and T2 generation plants (homozygous) were used for the further analysis.

## Slide 5
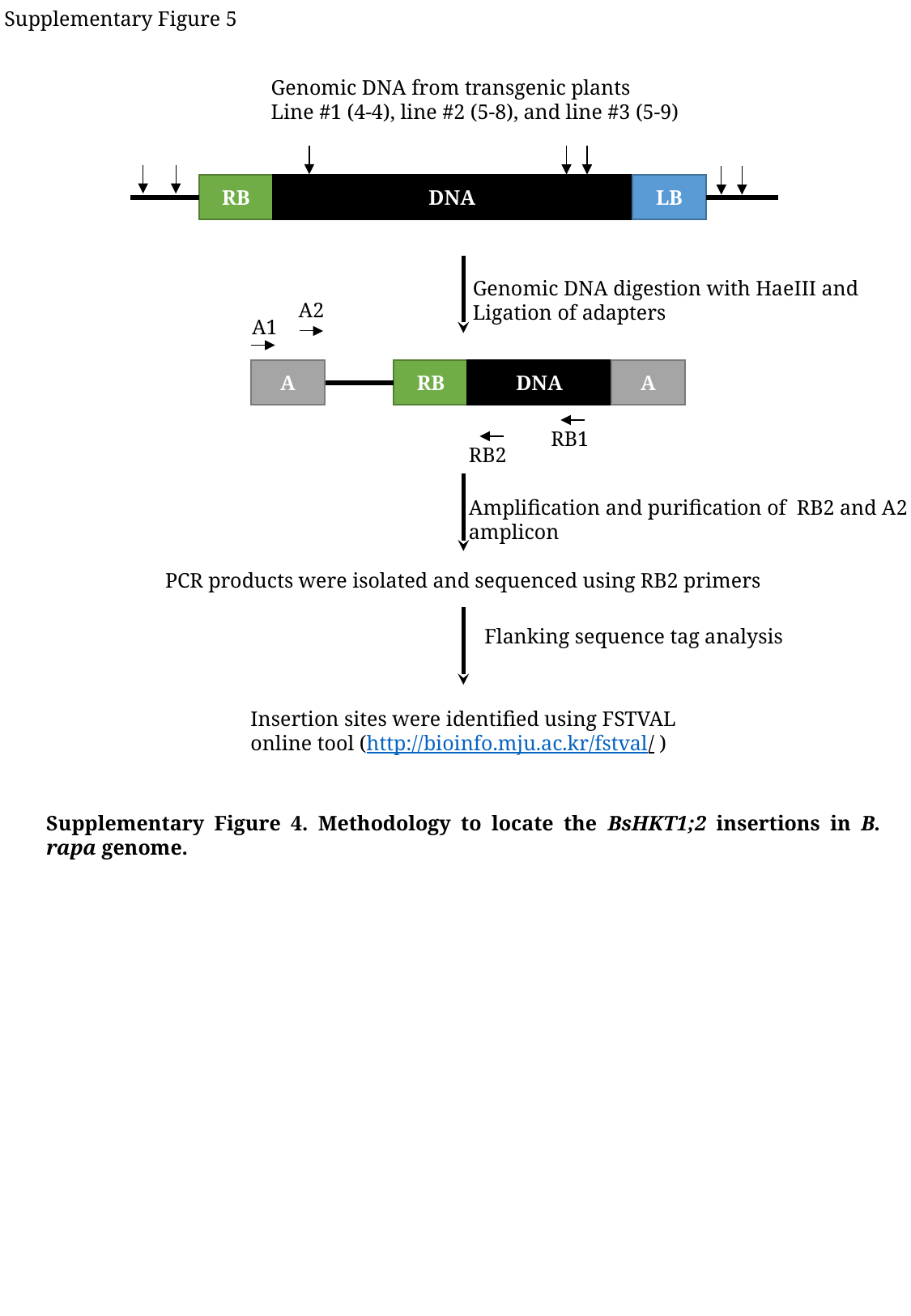

Supplementary Figure 5
Genomic DNA from transgenic plants
Line #1 (4-4), line #2 (5-8), and line #3 (5-9)
RB
DNA
LB
Genomic DNA digestion with HaeIII and
Ligation of adapters
A2
A1
A
RB
DNA
A
RB1
RB2
Amplification and purification of RB2 and A2
amplicon
PCR products were isolated and sequenced using RB2 primers
Flanking sequence tag analysis
Insertion sites were identified using FSTVAL
online tool (http://bioinfo.mju.ac.kr/fstval/ )
Supplementary Figure 4. Methodology to locate the BsHKT1;2 insertions in B. rapa genome.

## Slide 6
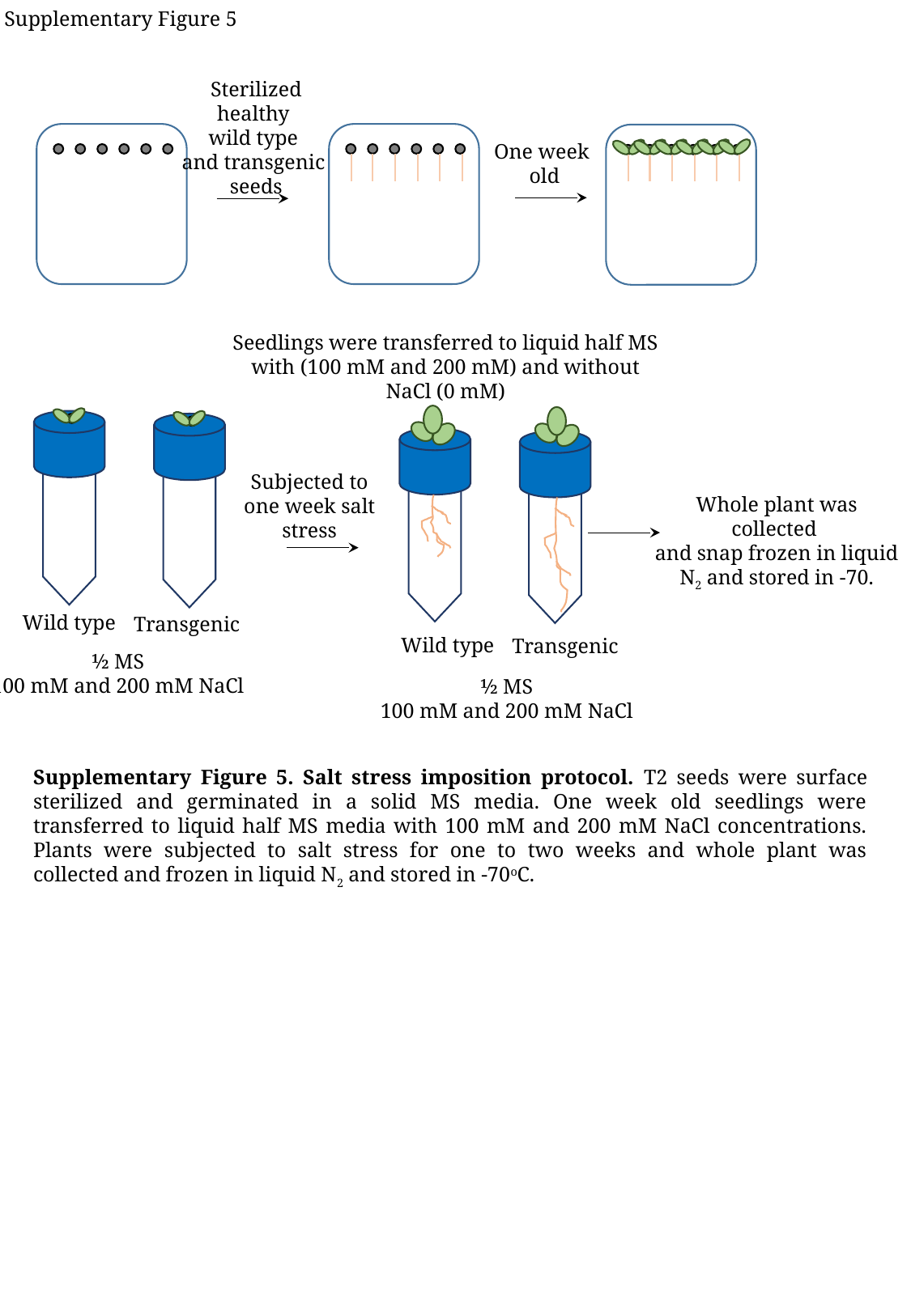

Supplementary Figure 5
Sterilized healthy
wild type
and transgenic
seeds
One week
old
Seedlings were transferred to liquid half MS with (100 mM and 200 mM) and without NaCl (0 mM)
Subjected to one week salt stress
Whole plant was collected
and snap frozen in liquid N2 and stored in -70.
Wild type
Transgenic
Wild type
Transgenic
½ MS
100 mM and 200 mM NaCl
½ MS
100 mM and 200 mM NaCl
Supplementary Figure 5. Salt stress imposition protocol. T2 seeds were surface sterilized and germinated in a solid MS media. One week old seedlings were transferred to liquid half MS media with 100 mM and 200 mM NaCl concentrations. Plants were subjected to salt stress for one to two weeks and whole plant was collected and frozen in liquid N2 and stored in -70oC.
