# Supplementary material for: Genome-wide identification of a novel Na+ transporter from Bienertia sinuspersici and overexpression of BsHKT1;2 improved salt tolerance in Brassica rapa
Source: Front Plant Sci. 2023 Dec 12;14:1302315. doi: 10.3389/fpls.2023.1302315 (PMC10773568; doi:10.3389/fpls.2023.1302315)
Supplement: Supplementary file 1 [file DataSheet_1.zip › Supplementary File 1.DOCX]

**Supplementary file 1. The Amino acid sequences of HKTs used for motif analysis and phylogenetic tree construction.**

>EcHKT1;1

MMSFFSSLGKEVAFLCSASWIKLACICRSLCFLLSCCFRFLLLRVNSFCIQVFYFVFLSFLGFWVLKALGPRTDSFRPRDLDLFFTSVSATTVSSMSTVEMEVFSNSQLVVMTVLMFIGGEVFISLVGLHLRKSKLRWRIRTEDKVASADGNLCPSAPTNDIVDHIELGVVAKTDCLNSQVEPQFYRPQDKSSDLDYLKYCSVRFLCYVVLGYLLVVQVLGVAAVSLYITLVPSARDVLKKKGLKMVTFSVFTTVSTFASCGFVPTNENMIIFSKNSGLLLILIPQALLGNMLFPSSLRLTLWLIGRFSEKDEIGYLLSRTSEIGYKHLLPSLYSSLLGVTVLGFVGIQFIMFCSMQWDSESLNGLSSCEKIVGALFQCVNSRHTGETVVDLSTVAPAILVLFVVMMYLPPYTSFLPVKGNERFPENGERRKPKQSYRLLLENLKFSQLSYLAIFIIVICITERKKMKEDPLNFNVLNIVVEVVSAYGNVGFTTGYSCERQLRPVKGCEDKWYGFSGKWSDESKIILIVVMFFGRLKKFNMKGGRAWILL

>EcHKT1;2

MMRFPCLGKEVVCLCIASCLKIARLFRSFCFLFSWYFRFLLLRVNSFCVQLLYFVFLSLLGFWVLKASRPLTHSFRPRDLDLFFTSVSLATVSSMSTVEMEVLSDSQLVVMTVLMFVGGEVFTSLAGLHFRKLKLQSLLKTEETVASVHTNLCPSNPTNGVVDHVELAVVTNSDCLNSRVEPQLHGPRDESPDLDYLKYRSANVLCLVVLGYLLGVNVLGVAAVSLYMILVSSARDVLKRKGLKMMTFSIFTTVSTFASCGFVPTNENMAVFNKNSGLLLILIPQVLLGNTLFPSTLRLVLWLLGKFSKKAEIDYLLSRTGEIGYKHLLPSLYSSLLVVTVLGFIGVQFIMFCSMQWDSESLDGLNSYEKIVAVLFQCVNTRYTGETIVNLSKVSPAILVLFVVMMYLPPYTSFLPAIGGEELLGNGERKKAKRSHKLMLKSLIFSQPSYLAIFIITICITEKEKMKKDPLNFNVFNIVVEVISAYGNVGFTIGYNCDRQLRRIEGCEDKWYGFSGKWSDEGKIILIAVMIFGRLKKFNMKGGRNWILL

>HvHKT1;1

MPELESPQTSMHRFSSALVILQNLPSHTAMKLPLSNLEVLRITKEKVKRFHEFVSTRLSSLSKCTADLFRRSYLFLVFKSNPLIVQLIYLLSISFAGFLALKNLRPLNKPSPRNLDLMFTSVSTVTVSSMATVEMEEFSGQQLWVFIILMILGGEVFASMIGLHFKNIRANTEEAFQTRLDFISRDIESSDVFTNSGQNNMEGAQSEETMPHNQVQESKAMNQKSRNVLAHVVAVYFIAAIVCSSVVITIFLWIDSDARHLLKSKHIKIWTFSIFTAVSSFANCGFTPLNDSMAIFKNNPTFLLLLTPQILVGNTLFAPLLRLSIWTLGKVSSREEYAYILQHPKEIGYRHLQPHKNSVKLVLTGVMLILLQAMLICYFEWDSKSLEGMGWFQKLTGSLFQSANSRHAGETVINISTLSPPIMVIFALAMYLPSGTSILASRGDNRSLADKKENPNGRATWKKFAMTKRTCLVIFTILACITERKSMTADPLNFSIFSVIFEVISAYGNVGYSLGYSCDKLVKPDATCRDASYGFVGRWSDQGRLIIILVMFLGRFKAYNLKGKKPLNVHPCTGATPHERPEVAAN

>HvHKT1;5

MGSLHVSGSTTTQHSRVQRAYQLLFFHVHPFWPQLLYFVSISFFGFVMLRALPMKTNMPTDLDLIFTSVSATTVSSMQAVEMESFSNPQLLLLTLLMLLGGEVFTSMLGMYFTYVKSKKKEAQAPHDDGAKVKPAPSSLELTAASICMDDGTAQDRMEQGFKDQPRYGRAFLTRLLLFIVVGYHAVVHPAGYSLMLVYLSVVSGARTVLAGKGISMHTFSVFTIVSTFANCGFMPNNEGMASFRSFPGLLLLVMPHVLLGNTLFPVFLRLAIWALQRFTKRPELGELRSIGYDHLLTSRHTRFLAFTVAVFVLAQLSLFCAMEWGSDGLRGLTAAQKLVAALFMSVNSRHAGEMVVDLSTVSSAVVVVYMVMMYLPPYTTFLPVEDSNQQVGTDQKRTSIWHKLLMSPLSCIAIFVVVVCITERRQISDDPLNFNVLSIAVEVISAYGNVGFSTGYSCGRQVTPDGSCRDAWVGFSGKWSREGKLALIAVMFYGRLKKFSMHGGQAWRIV

>McHKT1;1

MEKYLALFHKKLDQFFNFCYTKALSYISSLYEYIIFQVHPFWHHLFYYIIVSLLGYFSLKATSRKQRSSPVFSDPRHDDLDLFFTSVSATTISSMSTIEMEDFSSPQLVVLIILMLSGGEVFLSLLGLQLRKSKHRKREQKNILLNSPSEGMKYKSLVALGHVVLGYLLVCHIIGYSLASLYISISRSASNVLERKHIEMHLFSLFVTVSTFSNCGFIPTNENMMVFKGNSGLLLILIPQILLGNKLYPSCLRLVLWVLEKLTGKAEFSYILKKNDELGYGLFFSQVDALLLAVTAVGLVMVQFVVFCILEWNSVALMDLNWHQKLVGSLFQTVNSRHSGESIIDLSLISPATMVLFVVMMYLPSYTTFIPVGYDKEISPEMSSKKSKKQGCSIAENLKFSQLSYLAIFVMIVCITERRNLVEDPLNFNVFNIIVEVISAYGNVGFSMGYSCKRRLGDIGTCKDAGYGFVGRWSRQGKMVIILVMLFGRLKRFNFKGGKAWKLSL

>McHKT1;2

MGRFGFLKEKVQQLYGCLCVGLFYLLSTLFWVSNKIYDFITYRMSHFSIEVCYFIFVSCLGFLILRNIKPRTYPVKPGELDMFFTSVSAATVSSMATVEMEVFSDAQLHIMTILMFIGGEVFTSMVGLHFKASRLGNTPLGVKSRANSVASLPCPPEDFDHIELGIITTTTTTTTTTTTTLQKTKSEIDFLLKSRSIRVLGFVVLAYLLIVHVLGTIMVYAYLRIEPSAKRVLETKGLKSITFAIFTSVSTFSSCGFVPTNENMIIFRQNSGLLLMLIPQVLLGNTLLPSFLRLTIWVLGKFTKKDESKYLMRNTKEIAYHHLLPTKHSKYVVVTVFGFILASLIMFCSMDWNLKGLSDLNVYQKLVGALFQCVNARHTGETIVDLSTIASAVLVVFVIMMYLPPYTSFLPTKDGEEEYPLVYKGEKTKGKLILDNVVFSQLSYLVIFIILVCITERKSMKEDPLNFNVLNIVVEVISAYGNVGFSTGYSCSRQLKPDANCVDKWYGFVGKWSDQGKIILIFVMFFGRLKKFNMKGGRAWKLL

>OsHKT1;1

MHPPSLVLDTLKRIKLYIAMKLLLPNSEVPRIYWEKAQHLCGFLSMKLISRARCVASSVKQSYSFLVCKSNPLVVQLVYFVIISFAGFLALKNLKPQGKPGPKDLDLLFTSVSTLTVSSMATVEMEDLSDRQLWVLILLMLMGGEVFTSMLGLYFNNANANRNENSQRSLPSISLDIEFNSPANNGDHKITECGQSEETMSQNQVQQNKSITYNPCAVLVRIVTGYFVATVISSSVIIIIYFWIDSDARNVLKSKEINMYTFCIFTAVSSFANCGFTPLNSNMQPFRKNWVLLLLVIPQILAGNTLFSPLLRLCVWVLGKVSGKAEYAYILQHPGETGYKHLHVRRNSVYIVLSVTGLILLQVMFICSFEWNSESLEGMNWLQKLVGLLFQSVNTRQAGESILDISTLSPSTLLLFAVVMYLPSDASFLTANADNQPLTDKKTNSISRALWRNFTVNKLSCLAMFTFLACITERKSISSDPLNFNIFSIVFEIISAFGNVGYSLGYSCQKLLKPDATCKDASYGFVGRWTEEGKLIVILVMFLGRLKEFILK

>PtHKT1;1

MKSFACFGKKLEHPRNFFCNKVSCFHKSSMCSIRSFLRVLVFQIHPFWVQLAYFVILSLVGHMALKVSKPRPGSLRPASLDIFFTSVSSATVSSMSTVEMEVFSNTQLIIMTILMLLGGEVFTSILGLYLSRFKFSKHETKESRVSSVYHNPPKRTNFPGLEIEKPTNVDLECNLNSLDNDHSLKLNSLKSLACVALGYFSVVHITGSSLVAMYTSLVPSARQVLGSKGIKIQTFSVFTTVSTFSNCGFVPTNENMVAFKKNSGLLLILIPQILLGNTLYPSCLRFLIWILEKITRKVEFRYILMNTREMGYGHLLSFSHSCLLAITVSGFILVQFILFCSMEWNSGAKDGLNPYQKLMGALFQVVNSRHTGESIVDLSIISPAILVLFVVMMYLPPYTSFMPKKQQEEVDSETGQKCKNQRKSLVQCLLFSPLSTLAIFVILICVSEREKLKKDPLNFNVLNITIEVVSAYGNVGFSTGYSCKRQLEPDSSCKDAWFGFVGRWSNMGKFILILVMFFGRLKKFSINGGKAWKLS

>SabHKT1;1

MLSINNIVVNKYKHLCNSLYLIILHLFSSLHWISSKTCDFIIINISHFIIELCYFILVSSFGFLILKTLNPKSIHNNNNHIINPIKDLDLFFTSVSATTVSSMSTLEMEVFSNSQLIVLTILMFIGGEVFTSMIGLHFSASKLVYKPLKSRSRVNSVASLTLPCKPIELGLIVVTPQENNTSTTMQKTKSEIDFFIKSKSIRVLGFLVLLYLFTIHILGISMVLLYLNITPNAINVLHKKGIETFTFSIFTIVSTFASCGFIPTNENMQVFSKNSGFLLILIPQILLGNTLFPSFLRFSIWVLGKFAKKDEAKYLLRNAKEIGYHHLLPSKHSRLLIMTVLGFIMVQLVMFCAMEWGNEGINEGHNVYQKLVGILFQCVNSRHTGESIVDLSSIAPAMLVVFIVMMYLPPYTSFVPIKDKEKEYTHILCKEEDKSRKILKNVLFSQLSYLAIFTIIICIIEKQNMKDDPLNFNVFNIAFEVISAYGNVGFSTGYSCGKQLKGDPKCVDKWYGFAGRWSDQGKLVLILVMFFGRLKKFNLKGGKAWKLL

>TaHKT1;5-B1

MGSLHVSCSTTQHSKLQRVYQLLFFHVHPFWLHFLYFVTISFLGFVILKALPMKTSMVSRPIDLDLIFTSVSATTVSSMVAVEMESFSNPQLLLLTILMLLGGEVFTSMLGLYFTYIKSKKKEAPHDHGDGGGKVEPAPSSLELPATTFMDDSTAQNQMEQGFNKEQPRYGRAFLTRLLLFIVLGYHVVVHLAGYSLMLLYLSVVSGARAVLAGKGISLHTFSVFTVVSTFANGGFVPNNEGMVVFRSFPGLLLLVMPHVLLGNTLFPVFLRLAIWALRRVTRRPELGQLQSIGYGHLLTSRHTCFLAFTVATFVLAQLSLFCAMEWGSNGLHGLTAAQKLVAALFMSVNSRHTGEMVVDLSTMSSAVVVLYVVMMYLPPYTTFLPVEDDSDQQVGADQHHQKRVTSIWRKLLMSPLSFLAIFIAVVCITERRQISDDPLNFNVLNITVEVISAYGNVGFSTGYSCARQVTADGGCRDTWVGFSGKWSWQGKLVLIAVMFYGRLKKFGMHGGEAWRIV

>ThHKT1;1

MERVVDKLAKIFSQHAKSLPLFFLYFFYFLFFSFLGFLALKISKPRTTSRPHDLDLFFTSVSAITVSSMSTIDMEVFSNTQLIIITILMFLGGEIFTSFVNLYFSHFINFKIKHLVGSFNFDRPINDPGSDLENVTNHVKLSSQINERASKCLYSVVLGYLFVTNIAGSTLLLLYVNFVKTARDVLSSKKISPLTFSVFTAVSTLSDCGFVPTNENMIIFRKNSGLLWLLIPQVFMGDTLFPCFLVLAIWGLHKITNREELGYILKNHKKMGYSHLLSVRLCVLLALTVLGLVMIQFLLFCTFEWNSESLEGMNSYEKLVGSLFQVVNSRHTGETVVDLSTLSPAILVLFILMMYLPPYTLFMPLTVEKNKKEGEHDSGDEIKGKKNGFYVSQLTFLAICIFLISTTESQKLRRDPLNFNILNITFEVISAYGNVGFTTGYSCERRLDISDGSCKDASYGFAGRWSPVGKFILIIVMFYGKFKQFSAKSGRAWILYPSSS

>TmHKT1;5

MGSLHVSSNATQHSKLERAYQLLVFHVHPFWLQLLYFVSISFFGFVILKALPMKTSTVPRPMDLDLIFMSVSATTVSSMVAVEMESFSNPQLLLLTLLMLLGGEVFTSMLGLHFTYVKSKKKEAQAPHDHDDGDKGKPAPSCSLKLAATTCMDDVDRVEQGFKDQPRYDRAFLTRLLLFIVLGYHVVVHLAGYSLMLVYLSVVSGAGAVLTGKGISLHTFSVFTVVSTFANCGFVPNNEGMVAFRSFPGLLLLVMPHVLLGNTLFPVFLRLAIWALRRVTRRPELGELRSTGYDHLLTSRHTWFLAFTVAAFMLAQLSLFCAMEWGSDGLNGLTAAQKLVAALFMSVNSRHTGEMVVDISTVSSAVVVLYVVMMYLPPYTTFLPVEDDSDQQVGADQHDHKRITSICHKLLMSPLSCLAIFIAVVCITERRQISDDPLNFNVLNITVEVISAYGNVGFSTGYSCGRQVTPDDGDCRDTWVGFSGKWSWQGKLALIAVMFYGRLKKFSIHGGQAWRII

>VvHKT1;1

MKKFPCFARKPVDVFNRSSTKLSCLQQSFRGLFSSLFYFLAFQVSPFWNQLGYFITVSLLGYMALKVSKPKTTSFMPSDVDVFFTSVSASTVSSMSAVEMEVFSDTQLVIMTVLMLVGGEIFTSMLGLQFVRSKYTRKANRENKAHLASIDYKSPNSKISFDQIELGLVTLPQAQNEQPCSNLEKGIEASCDEDLKYHSIKCLGYVVLFYLLVVHVVGSALIVLYLNLVPSAREVLKNKGLRILTFSVFTVVSTFSNCGFIPTNENMVVFKKNSGLLLILIPQILLGNTLFAPCLRFVIWVLVKITRRVEFNYMLKNSREISYDHLLPGLYSCLLAITVFGFILVQFLLLCFMEWNSEDLAGLNAYQKIVGMLFQTANSRHSGESIFDLSVISPAVLVLFVLMMYLPPHTSFLPIDGGEKALQKEERRTEKRKYVEHLLLSQLSYLVIFVILVCIIEREKMKKDPLNFSVLNITIEIVSAYGNVGFSAGYQCNLQLKHEPHCKDLCYGFVGRWSNSGKFVLMFVMFFGRLKKFSMHGGRAWKLD

>VvHKT1;2

MWIHLSYYITLSLVGYLALKVSKLKTTSFRSSGLDLFFTSVFASTVSSMSTMEMEIFSNIQLVIMTILMLLGGEIFTSFLGLQLLRSKYFSKRENIETRVHVNASNVEKFVNQVELDLATLPQSQNEKPDPNLENGSGDSNCVIFCYILVVHLVGSALILLNLRLVPSAREVLNKKGLSIQTFSIFTTVSTFSNCDFIPTNENMMIFKKNSDLLLLLLIPQKRSKRVEFVGYMSKNSREIGYSHLLPSLHSSFLVITVFLMILVQLILFGSMEWNSEVVEGLNTKQKLVASLFQVVNSRHTGESVFDLSTISPAVLVLFVVMMYLPAYTSFLPIHDCQKALKDRKRRTKRNKLLEYLPFSQLIFGALHHSHLDKMREDPLNFNVLNVTIEVVSAYGNVGFSTGYSCARKLKPDPSCKDAWYGFVGRWSDKES

>SsHKT1,1

MLSFKFIVEKCKHFYTSLYLLLVYVFTSLYWLISKIYDFIMVYVCHLIIELCYFILVSSFGFLFLKTLIPKSSNNNNNNNSINDLDLFFTSVSATTVSSMSTLEMEVFSNSQLIVLTILMFIGGEVFTSMVGLHFSASKLVYTPLQARSRVNSVASLPLPPESIELGVIIPSSTTTQEIRVSSSTIEKTKSEIDFLIKSKSIRVLGFVVLSYLIIVHFLGISMVLTYINTIPNAKNVLDKKGLKTFTFSIFSVVSTFASCGFIPTNENMQVFSKNSGLLLMLIPQILLGNTLFPSFLRFSIWMLGKFVKKDETKYLMRNSKEIEYHHLLSSKHSRFLIVTVLGFILVQFIMFCSMEWNFDGLNNDHNIYQKLVGILFQCVNSRHTGESIVDLSSINSAMLVIFIVMMYLPPYTSFLPIKDEEKEYPNMVLFNREKKRRKILKNFLFSQLGYIAIFIIIICITEKQKIKDDPLNFNVFNIAFEVISAYGNVGFSTGYSCEKQLKGDPNCVNKLYGFLGSWSDEGKLVLIIVMLFGRLKKFNLKGGKAWKLL

>BvHKT1,8

MQLLPHTHKMMEKYLVFLHEKLDDLLKIYHKKVEPFFSHLFEYLLFQINPYWHHLFYYILVSLLGYISLKGSKQNSSVSSNPHYDLDLIFTSVSATTISSMSTIEMENFSSTQLGVLVILMFSGGEVFLSLLGLQIRKLKHKRRARNHVLNPSPASEGDGMKFRSLRALNHVVLGYLLVSHILGYSLVSLYFSIVPSASNVLARKKIETHFFSLFTTVSTFANCGFIPTNENMIPFKKNSGLLLILIPQILMGNKLYPSCLRLVIWVLEKVTKKEEYSFMLQNHGGLGYGNLTSSYKAFLLGITAIGLVMVQFVVFGILEWNALVLEGQNGYQKFVGSLFQTVNSRHSGESIVDISLVSHATLVLFVVMMYLPSTTTFVPISYNKESALIENSSQSQTSRSSKKQGSSILENLKFSPLSYLAIFVILVCITESKSLKEDPINFSVFNIIVEVVSAYGNVGFSMGYSCKRRLENFSNCKDSWYGFAGRWSWEGKFLLILVMLFGRLKRFHFQSGKAWKLSL

>SeHKT1;2

MKLQQDLQKMMEEYIAFVLETSDKIKLFFQKRVSPFLSHSFEYLLFQISPYWHHLFYYILVSLLGYISLKGTNQRYYSPPSSSSSSLSSSYNAKKPFYDKKHHNLDLLFTAVSATTISSMSTIEMENFSNPQLMVIIILMFSGGEVFLSLLGLQIRKLKHKKRARTHVLDPRPVSQEEGMKYKSLRVLNHLVLGYLLVFHIIGYSLLSLYISVESSAGNVLTRKKLELHLFSIFTTVSTFSNCGFIPTNENMIVFKRNSGLLLILIPQILLGNKLYPCGLRLVIWVLERFTKKQEYSYLLKNHKGLGYGLLTSNYKAVLLGITSIGLVVVQFVVFSILEWNSLVLEGLSFYQKFVGSLFQTVNSRHSGESIIDVSQVSPATMLLFVVMMYLPSQTTFVPIRYDKESTIIIEEKSSQGSKRQKSALIENLKFSPLSYLAISIMLICITESRLLREDPLNFSVFNIIVEVVSAYGNVGFSMGYSCKQRLETNKPCEDRYFGFAGRWSWQGKFILILVMLFGRLKRFHFNSGKAWKLSL

>SeHKT1;1

MLSINNIVVNKYKHLCNSLYLIILHLFSSLHWISSKTYDFVIINISHFIIELCYFILVSSFGFLTLKTLNPKSIHKNDKININNPIKDLDLFFTSVSATTVSSMSTLEMEVFSNSQLIVITILMFIGGEVFTSMIGLHFSASNLVYKPLKSRSRVNSVASLTLPFEPIELGLIVVTPQENNTSTPTMQKTKSETDFFIKSKSIRVLGFLVLLYLFTIHILGISMVLLYLNITPNAINVLHKKGIETFTFSIFTIVSTFASCGFIPTNENMQVFSKNSGFLLILIPQILLGNTLFPSFLRFSIWVLGKFAKKDEAKYLLRNAKEIGYHHLLPSKHSRLLIMTVLGFIMVQLVVFCAMEWSNEGINKGHNVYQKLVGILFQCVNSRHTGESIVDLSSIAPAMLVVFILMMYLPPYTSFVPIKDEEKEYTHILCKEEDKRRKILKNVLFSQLSHLAIFTIIICIIEKQNMKDDPLNFNVFNIAFEVISAYGNVGFSTGYSCGKQLKGDPKCVEKWYGFAGRWSDQGKLALIIVMFFGRLKKFNLKGGKAWKLL

>HvHKT2;1

MGWVKRFYQDFIHIKLHSFCRISRYVVDSIAFVYRFVALHVHPFWIQLSYFLAIAILGSVLLMSLKPSNPDFSPPYIDMLFLSTSALTVSGLSTITMEDLSSAQIVVLTLLMLVGGEIFVSLLGLMLRVNHQDMPDLPRVKISSVPVELEEIDLANSMALSDESQLEEATHAITPKKCTGLKRSRSVKCLGYVVFGYFAVIHILGFLLVFLYITRVPTASAPLNKKGINIVLFSLSVTVASIANGGLVPTNENMVIFSKNSGLLLLLSGQILAGNLLFPLFLRLLVWFLGRLTKVKELRLMIKNPEEVHFGNLLPRLPTVFLSSTAIGLVAAGVTMFSAVDWNSSVFDGLSSYQKAVNAFFMVVNARHSGENSIDCSLMSPAIIVLFIVMMYLPPSATFAPPDGDIKTTNENTKAKRGSLVQKLAFSPLGFNIIFVIVACITERRRLRNDPLNFSTLNMIFEVISAYGNVGLSTGYGCSRLHQLHPEIICQDKPYSFSGWWSDGGKFVLILAMLYGRLKAFTMTMGKSWKV

>HvHKT2;4

MPIRLRTFLSSARHVSNSSVFIFQFIPFHLSPLLVHLSYFVIIDVLGFVALMALKPSNPNYSPRYVDIFFLSTSAVTVTGLATIKMEDLSSSQVVILTLLMLLGSEMFVSLIGHIHELRKQNKHDPEDSRVRSVTVQDESQIEEAIPATQSISTTSLKKSCLKYIGFVLLAYMVLILLVGSLSVFLYVAHVSTARDVLTRKSINTMLFSISVTVSSFTNGGLIPTNESMAVFSSNQGLLLLLTGQILAGNTLLPVFLRLVIWALRGLRIGRAKPEEFKFMMNNTKAVGFNHLLPNQQTVFLAASVAALIAVTVTFFCCLNWDSPVFAGLTANQKITNALFMAVNTRQAGENSIDCSLVAPAALVLFITMW

>OsHKT2;1

MTSIYHDFIHNKLQSFGRIGRYFVNFVVLAHRFIALHIHPFWIQLSYFLLISILGSVLLMFLKPSNPEFRPGYIDMLFLSTSALTLSSLITIEMEVLSSSQIVVITLLMLLGGEVFVSFLGLMLRLNHKHNPEFSGDKVSSVPIELDTINSASTVISCEELQLEAAIPEVPSSTIKDLKRSKRLRWFLGFVVFSYFVVIHVAGFLLVLWYISRVSSAKAPLKKKGINIALFSFSVTVSSFANVGLVPTNENMAIFSKNPGLLLLFIGQILAGNTLYPLFLRLLIWFLGKVTKLRELKLMIKNPEELQYDYLLPKLPTAFLASTVIGLMASLVTLFGAVDWNSSVFDGLSSYQKIINALFMAVNARHSGENSIDCSLIAPAVLVLFIILMYLPPSTTFALSNGDEKTANKKAKRKLGLVVQNLAFSQLACISVFVIVAFITERSRLRNDPLNFSALNMIFEIISAYGNVGLSTGYSCSRLQKLHPGSICQDKPYSLSGWWSDEGKLLLVFVMLYGRLKAFTKGTGEYWRLW

>PhaHKT2;1

MEAFHYEFIHSKMHSFICISRYVLDLFVFVYRFVASHLHPFFIQLSYFLVIDLLGSVLLISLKPNNPDFSPRYVDMLFLSTSALTVSGLSTVKMEDLSSTQIVVLTLLMFVGGEVFVSFLGLMLRPNHQAKPTDPAGNNKVSSIAVELETIDTASAIICEELQLEEEMHATPSLSSNDLKKSKSVRYLGFVVFGYLAVIHVLGFLLVFLYITHVPTARAVLTKKGINVALFSVSVTVSSFANGGLVPTNENMAIFSKNAGLLLLLTGQVLAGNTLFPLFLRLLIWFLGRVTKLEGLELMIRNPKELRFRHLLPKLLTAFLSSTVVGLAALAVVLFSVIDWNSLVFDGLSSYQKIINALFMAVNTRHAGENSIDCSLISPAVLVLLIVMMYLPSSTTFAPPNRDDKRKDDKVVPKRRSLVQNLAFSQLGCNIIFVMVVCITERRRLRNDPLNFSTLNMIFEVISAYGNAGLSTGYSCSRLQQLHPESICHDKPYSFSGWWSNEGKLMLIFVMLYGRLKAFTMGTGKSWKLG

>SbHKT2;1

MPIRFHVLASAARHAVSSSVLVCRLIAYHLTPLLLHLTYFLAVDLLGFLALVLLKPSNPGYRPRYIDVFFMSTSAVTVTGLATVEMEDLSSAQLVVLTLLMLLGSEMFVSLLGLVLESSRNRRQQQRDHQDHDSRVMAAAAVRDEPDLEEANGPAAAPSADSSGDGGDRNRKESSRDVRSLALVVSAYMAAILVVGSVLVFAYVATVPTARDVLARKRLSAALFSVFATVSSFTNGGLLPTNESMAVFAPNRGLLLLLAAQILAGSTLLPVFLRLVVSATRGLARALSLFTGRGGSVEELVPMDMEKSAAAAGFGHLLPSGSRAASLAATVVAVATAAAALLCCLNWNSAVFAGLTTGEKLTNAVFMAVNVRQAGENSVDCSLVAPAVLVLFLAMTCIPASATLLSVQDDGGEKTRSGAGEPERKDGAEKKRRRRLSLNSMLLSPLACNAAAVMLACITERRSIAGDPLNFSTFNVIFEVISAYGNVGLSTGYSCSRLPPPPATTACHDKPYSFSGWWSDQGKLLLVLLMLYGRLKGFHGQRRRR

>TaHKT2;1

MGRVKRFYQDFIHIKLHSFCRISGYVVDSIAFVYRFVALHVHPFWIQLSYFLAIAILGSVLLMSLKPSNPDFSPPYIDMLFLSTSALTVSGLSTITMEDLSSSQIVVLTLLMLIGGEIFVSLLGLMLRVNHQDMQDLPSVKISSVPVELEELDLPNSMALCDESQLEEAAHAIPPKKCTELKRSRSVKCLGYVVFGYFAMIHVLGFLLVFLYITHVPTASAPLNKKGINIVLFSLSVTVASCANAGLVPTNENMVIFSKNSGLLLLLSGQMLAGNTLFPLFLRLLVWFLGRITKVKELRLMINNPEEVRFANLLARLPTVFLSSTVVGLVAAGVTMFCAVDWNSSVFDGLSSYQKTVNAFFMVVNARHSGENSIDCSLMSPAIIVLFIVMMYLPSSATFAPPSGDTKTTNENTKGKVKRGSLVQNLAFSPLGCNIIFVMVACITERRRLRNDPLNFSTLNMIFEVISAYGNAGLSTGYSCSRLHQLHPEIICQDKPYSFSGWWSDGGKFVLILVMLYGRLKAFTLATGKSWKV

>AtHKT1;1

MDRVVAKIAKIRSQLTKLRSLFFLYFIYFLFFSFLGFLALKITKPRTTSRPHDFDLFFTSVSAITVSSMS

TVDMEVFSNTQLIFLTILMFLGGEIFTSFLNLYVSYFTKFVFPHNKIRHILGSYNSDSSIEDRCDVETVT

DYREGLIKIDERASKCLYSVVLSYHLVTNLVGSVLLLVYVNFVKTARDVLSSKEISPLTFSVFTTVSTFA

NCGFVPTNENMIIFRKNSGLIWLLIPQVLMGNTLFPCFLVLLIWGLYKITKRDEYGYILKNHNKMGYSHL

LSVRLCVLLGVTVLGFLIIQLLFFCAFEWTSESLEGMSSYEKLVGSLFQVVNSRHTGETIVDLSTLSPAI

LVLFILMMYLPPYTLFMPLTEQKTIEKEGGDDDSENGKKVKKSGLIVSQLSFLTICIFLISITERQNLQR

DPINFNVLNITLEVISAYGNVGFTTGYSCERRLDISDGGCKDASYGFAGRWSPMGKFVLIIVMFYGRFKQ

FTVKSGRAWILYPSSS

>BsHKT1;1

MLNFNFIVENCKQFYTSFCLLFAYIFTSLYWLSSKIYDFIIIYVSHFIIELCYFILVSSF

GFLFLKTLNPRSTHNNHPIINDLDLFFTSVSATTVSSMSTLEMEVFSNSQLIVLTILMFI

GGEVFTSMVGLHFSASKLVYTPLHSRSRVNSVASLPLPSEGIELGIIIPSSNEASSIEKT

KSEIDFLIKSKSIRVLGFIVLSYLFIVHFLGISMVLAYINTIPNAKNVLDKKGLKTFTFS

IFTIVSTFASCGFIPTNENMQVFSKNSGLLLILIPQILLGNTLFPSFLRFSIWVLGKFAK

KDETKFLMRNSKEIGYHHLLPSKHSKFLVVTVLGFILVQFIMFSSMEWNIEGLDGHNIYQ

KLVGMLFQCVNSRHTGESIVDLSSIASAMLVMFIVMMYLPPYTSFLPIKDEEKEYPNMLG

LCKGEKKRRKILKNILFSQLSYIAIFTIIICITEKQKIRDDPLNFNVFNIAFEVISAYGN

VGFSTGYSCGKQLKADPKCVNKWYGFAGSWSDEGKLVLIIVMIFGRLKKFNLKGGKAWKLL

>BsHKT1;2

MELQLYLLKIMEKYLALLHENSDKIKVFFQKKVSPFFSHGFEYLLFQISPYWHHLFYYIL

VSLLGYISLKGTKQSYSSPKKAIYNPQHHDLDLFFTSVSATTISSMSTIEMEKFSNAQLM

VIILLMLSGGEVFLSLLGLQIRKLKHKKRARNHLLNPNPASQEEGMKYRSLRALNHVVLG

YLVVSHIIGYSLLSLYISIDSSASNVLETKKLEIHLFSIFTTVSTFANCGFIPTNENMVV

FKRNSGFLLILIPQILMGNKLYPCCLRLVIWVLERLTKKEEYSYLLKNHEELGYGLLTSN

YKAFLLGITSIGLVIVQFVVFSILEWNSVVLQGLSLYQKIVGSLFQTVNSRHSGESILDI

SQVSPATMLLFVVMIEQEPSSKQKAKE

>BsHKT1;3

MKASITTIFHHYIIPLINPFSLHLCYFLVLSLAGFLSLKVSKTRTSETPSNLDLFFTSVS

AATASSMTTVEMEVFSNDQLIVMTILMLLGGEVFTSMLGLHLRSCEFPSIQNPKLESSCS

IDSIEYNKNKSIKLLGYVVLGYIIIVHLVGSTLITMYMSLTPSALNVLNNKGLVLQTFSF

FMVVSTFSSCGFAPTNENMMIFRMNNPGLLLILLPYTFVGNTMYPLFLRLVIWVLEKLSR

KKEFNYILKNYEELEYGHLMSSKKCWYLGGTTIVFLVLQIVVFCGMDWSSQVMEGMSSYE

KFVASLFQTANTRHSGESVVDISQLSQAVLVLFTIMM

>BrHKT1;1

MDRVAGRKFCKIRSKLYKNRPCFLNFIYFLSFSFLGFLALKVFKPRATSRLHDLDIFFTSVSAITVSSMS

TVDMEVFSNTQLIIITILMFIGGPIFTSFFNIYLSHFTKFVFPQSKIKHLIGSFKADHTIEDRHLDQENI

NDRHEIPSQINEKASKCLYLVVIGYNLVTNIAGSMLLLVYVSFVKTARDVLRSKEISTLTFSIFTTVSTF

ATCGFVPTNENMIIFRKNSGLLWLLIPLVLMGNTLFPCFLRLLIWGLSKTTKREEFGYILKNRKKMGYSH

LLSVRLCVFLGLTVLGFILIQLFLFCTFAWSSESLAGMNWYEKLVGSLFQVVNSRHTGETIVDLSTLSPA

ILIVFILMMYLPPYTLFMPFTIKKKNKKEEENDSGYEKGGKKSGLLVSQLSFLVICIFLISITERQKLRR

DPLNFNVLNITLEVVSAYGNVGFTTGYSCERRLNVSDGGCEDAGYGFAGRWSSSGKFILIIVMFYGRLKQ

FTAKSGRAWILYPSSS
