# Supplementary material for: Genome-wide identification of a novel Na+ transporter from Bienertia sinuspersici and overexpression of BsHKT1;2 improved salt tolerance in Brassica rapa
Source: Front Plant Sci. 2023 Dec 12;14:1302315. doi: 10.3389/fpls.2023.1302315 (PMC10773568; doi:10.3389/fpls.2023.1302315)
Supplement: Supplementary file 1 [file DataSheet_1.zip › Supplementary File 2.DOCX]

**Supplementary file 2. Gene sequences of BsHKTs.**

>AtHKT1;1

AAAAAACAGGAATCGCTATCATCAGTAATAGTCATCATTAATCAATTTATATGTAATATGTGGCTGACAA

TTTCCATGTACGTGTAATATGTAATATATAAACACAACTTATGGCCAGTATAATATTAATGCTTAAACCG

ACTCGAGAACTAAAATGGACAGAGTGGTGGCAAAAATAGCAAAAATCCGTTCGCAGCTTACTAAATTACG

TTCACTATTCTTCCTTTACTTCATCTACTTCTTGTTCTTCTCCTTTTTAGGGTTTTTGGCACTCAAGATC

ACAAAGCCAAGAACCACTTCACGTCCTCATGACTTTGACCTTTTCTTCACTTCTGTCTCTGCCATCACCG

TCTCTTCCATGTCTACCGTCGACATGGAAGTCTTCTCCAACACCCAACTTATCTTCCTCACTATCCTCAT

GTTCCTCGGTGGCGAAATCTTCACCTCCTTTCTCAACCTCTACGTCTCCTATTTCACCAAGTTCGTCTTC

CCTCATAACAAGATTAGACATATTTTGGGATCTTATAATTCGGACAGTTCCATCGAGGATCGCTGTGACG

TTGAGACTGTTACTGATTATCGCGAGGGTCTTATCAAGATCGATGAAAGGGCATCTAAGTGCTTGTACTC

GGTGGTTCTTAGTTACCATCTTGTTACTAACCTAGTTGGCTCTGTGTTGCTTCTTGTGTACGTAAATTTT

GTTAAAACGGCGAGAGATGTTCTTAGTTCCAAAGAAATCTCACCTCTCACTTTCTCCGTCTTCACAACTG

TTTCCACGTTTGCAAACTGCGGATTTGTCCCCACGAATGAGAACATGATCATCTTTCGCAAGAACTCTGG

TCTCATCTGGCTCCTAATCCCTCAAGTACTGATGGGAAACACTTTGTTCCCTTGCTTCTTGGTTTTGCTC

ATATGGGGACTTTATAAGATCACAAAGCGTGACGAGTATGGTTACATTCTCAAGAACCACAATAAGATGG

GATACTCTCATCTACTCTCGGTTCGTCTATGTGTTCTTCTTGGAGTGACGGTGCTAGGGTTTCTGATAAT

ACAGCTTCTTTTCTTCTGCGCCTTTGAATGGACCTCTGAGTCTCTAGAAGGAATGAGTTCGTACGAGAAG

TTGGTTGGATCGTTGTTTCAAGTGGTGAATTCGCGACACACCGGAGAAACTATAGTAGACCTCTCTACAC

TTTCCCCAGCTATCTTGGTACTCTTTATTCTTATGATGTAAGTTTCTTTCAATCCTCTCCTCATATATCG

AATCATTTGCATGAACTCGAAATATTTAGATCGAAAGGTGCACTTATTTCGACATAATTTTCACATATGA

GGTGATATGCATGCACACATAGGTGCAGTTTTACGAATACATTAAACTAAAACAATTGAGATATAGTGAA

ATTAAATGGTTGACATTTCACTACAGAAACAATAAAAAGTCACCGCATGAATTAAAGTTGGTTGACATTT

CACAACAGAAACACCAAACATATTATTATCTCACGTAATGTAGTTTGGAAAATTATCAAATCATTTACCT

AATTTTTTTTTTTATGAAGCAAACATTTTCCTAACTATTATTGTTTATTCCATACCAAAAAAAAAAACGT

TTATGATTTATTTAAGGATTTTTTTAATTACTACGTAAAGGCCAATCATGCATGAGTGGGGTGGTAGAAC

CATAGATTTGCATTAGTTTTAGTGCTATTCTTTTGGCTGAATCCACCAATTAATCAGCTTAGCAATTGAA

AGCATATTTTATTAACCAAAATTAATTTTAACATATCTAACGTGGTCCATTTGGTTAAAGTATTCACTCC

CATGGATTGAGGAAGTAAGATTTAAGTTATAACCATGCTTGGCAATTATACATTTAATGAATAGTTTGAA

TACCAAGCTAATTTATAATTTTTGGAGATTAATTAGTATTTATTTCGGTAAATCATTCTTGTAACAAAAA

AAATACTAATAATATAAAAAATTGAGATTGTGAAGTTTATTTTTTCACCACAAACACAAATTTCATAGAA

TTTTTCAAGGTTTCCACAAATTAGTTTACGCTCTTAAAATAACAAACAAAACAAAAAGTAGTATGCTTGT

CAAACTTTCCACAAAAACATTAACGCCGGAAAACTTAAAACAAATAGTCATAATACATCTTTATTAAATA

AATAAAAAGAAGGCATAATACATATATAAGTTGATAAAACTCTCCATCACTTAAGAGTAATTTTTTGATG

TTTTCACTTAGAAGAATTTATATATATCTATATTAACCATTAATTAAGGAACCTACAAGAGAATGTGGTA

GCTAGACAAGATTTGATAGAGTGAATCAGAATATATGTCCATGGTTTTAGATTCAGTAAAAAAAAAATGT

CCCTTCAATGTAGTGGAGACTGGAGAATGTCCCTTCATCATATTCACAAAGCATTATTCTCTGTTTTTCC

ATAAAAACTTCTTTGGACTAATTAAAAAGTAAAATATAATTGCGTTTCCAAGGTATCTTCCTCCATACAC

TTTATTTATGCCGTTGACGGAACAAAAGACGATAGAGAAAGAAGGAGGAGATGATGATTCCGAAAATGGA

AAGAAAGTTAAAAAGAGTGGACTCATCGTGTCACAACTTTCCTTTTTGACGATATGTATCTTTCTCATTT

CAATCACCGAAAGGCAAAATCTACAACGTGATCCGATAAATTTCAACGTCCTTAACATCACTCTCGAAGT

TATCAGGTATGTTTCTATATCTCAAGATTCTTTAACCAAAACCAAATAAAAAAATTGTTTCAGCAATATA

TAAATCTCTATATTACATATCATGCATCCATAATAACTAAAAATTTCAAAATATTTTCAAACAAAATACG

TCATCTAGGTGATACATTATTCTACAATTTGACAACCCAAGTATTCGTAATTCACCTAACAAAAAAAGAG

GACGTGAATAAAATCGTATAAGAAACATATATACAGTAAAATCTCTATAAATTAATAATGTTGGGACTGC

AAAATTTTATTAATTTAGAGAGATTTTTTACTTATCGATAAATTAATAAATTAATAGAGAGATCTTCATA

ATTTAATAACTTTGAAAAAATTTCTCATTATAGAAAATATCTTTCTAAAATCAATTACAAGTAAAAAATA

ATATCATGTATAGAAAACAACACCAATATGTTTTGATATAGTAAAATATTAGAACTAAATTTCAAATAAG

AATATATACAAATTTAAGCAAAAAAATATCAGAAATATATTTTTAAGAAATTTCTTACATATAATGTATA

TATATAGTTGATATATTTGTGCAATTATTAATTTCTGATATTGATGGACCATATATTTACATAGGACTTT

CAAAAAAATTATTATCTAATTAATTTATCGATTTATGTCATTTTTTATACTACTCCCAACTCGGGACCGG

AAGAATTTATTAATTTATAGAGGTTATTAATTTATCGAGTATAAATTTATAAAATAAAAAGAAACATATA

TATATATATAATATATATATATATATATAATTCACTCATTATGAATTGATTGTGAATTAGAAGTTAAGAA

TTATCAACAAAATAAGTTTTGTAATGGCAGTGCATATGGAAACGTTGGTTTCACTACCGGGTACAGCTGT

GAACGGCGTGTGGACATCAGCGATGGTGGCTGCAAAGACGCGAGTTATGGGTTTGCAGGACGATGGAGTC

CAATGGGAAAATTCGTACTAATAATAGTAATGTTTTATGGTAGGTTTAAGCAGTTCACAGCCAAATCTGG

CCGCGCATGGATTCTTTACCCCTCGTCTTCCTAACATATTTCGATCATATATAAATGTTATATTAAAATT

AGTCACTTTTAGCATTCCTTTTCTGTGAGTGATGTACAATAAGTTTGTATGTAGTTTGCATCATCGTTAA

TCCTCGTTAATATTATGATAATGTATGAAGATATAATATAAGGAGTATTGTGTGTCATCTTGTGTTTCCT

GTTGTAACTTGTAATTCGAAAAATTAAACTTTATCTGTTTGATATGTATCGTACAAATAGTAACCAATCA

GTAATATAT

>BsHKT1;1

TATATAAAGGCAGCTAATTAAGAGTCTAAGACCAAACAAGTCCTTACAAAACCAAAAAACAAAACCCCTTTAAGCTAAACTTAGTCCATACTCCACTATCAAAAGATGTTGAATTTCAACTTTATTGTAGAAAATTGTAAACAATTTTATACTTCTTTTTGTCTACTTTTTGCCTATATTTTTACATCCTTATATTGGTTATCCTCAAAAATCTATGATTTTATCATCATTTATGTTAGCCACTTTATAATTGAACTATGCTACTTTATCCTTGTATCTTCTTTTGGATTCTTGTTTTTAAAAACCCTAAATCCAAGATCAACCCATAATAATCACCCAATAATTAATGATTTAGATCTATTCTTCACCTCAGTTTCAGCCACAACAGTTTCAAGCATGTCAACCCTAGAAATGGAGGTATTCTCAAATTCCCAACTAATTGTTTTAACCATTTTAATGTTCATAGGAGGTGAGGTCTTTACCTCCATGGTAGGTCTCCATTTTTCGGCCTCGAAACTTGTATATACACCCTTACATTCAAGAAGTAGGGTTAATTCAGTTGCTAGCTTACCACTTCCTTCTGAAGGTATTGAGTTAGGAATCATTATCCCATCATCAAATGAAGCTTCTTCAATTGAAAAAACAAAATCAGAAATAGATTTCCTCATAAAATCTAAATCAATTAGGGTTTTAGGTTTCATAGTTTTGTCTTACTTATTCATAGTTCATTTCCTAGGAATTTCCATGGTATTAGCATACATTAATACTATCCCAAATGCCAAAAATGTTCTTGACAAAAAAGGTCTTAAAACATTCACTTTTTCAATTTTTACAATTGTTTCAACTTTTGCTAGTTGTGGTTTCATCCCTACTAATGAAAACATGCAAGTTTTTAGCAAAAACTCTGGCCTTTTATTGATTTTAATCCCTCAAATTCTACTTGGAAACACATTATTTCCTTCATTTCTTCGATTTTCGATATGGGTATTAGGAAAATTTGCCAAAAAAGACGAAACTAAATTTCTAATGAGAAATTCAAAGGAAATTGGGTACCATCATTTGCTTCCTAGCAAACACTCAAAGTTTTTAGTAGTAACAGTTTTGGGGTTTATTTTGGTGCAATTTATAATGTTTAGTTCAATGGAATGGAATATTGAAGGATTAGATGGACATAATATATACCAAAAATTAGTGGGAATGTTATTTCAATGTGTTAATTCAAGACATACAGGTGAAAGCATTGTTGATCTTTCCTCAATTGCATCAGCTATGTTGGTCATGTTCATCGTTATGATGTTAGTTTTCTCCTCTCTCTTTTTTTTTTTTTTTTTTTGAATTTTTGAATTTTTTTTATATATTATTTTTGTCATTGCAAATTATTGACATGTTAACTGTTAGTCTGCTACGATGCATGTGAGTCCTTGCATGTGCATGCATATGTGTTATGTTTGTGTTTTTCTACATTTAAATTGTGAGCATGTCTTGACTCTTGCGTAAGATTATTGTTGTACGGTGATACTATAATTGATGATGGATTTATTATGGGATACATAAGAATATTGGATAATGTAACAGATGATTTTTACATAGCTTTTTATCTTTGTATATACACATAGTACATCAATACATCATATTAGATTAACGTTGAAAATACGAGTAAAAGTACGATTGAATTTAAAGTCAAATACTTTTGTCAGACGGTTTGATATTAGACTATTTAGGTATTATACTTAAGAATAAGTGATAAAAGTTAGGCAAAATTTGTTGGAACAGCACCACCTATTGGTCAGGTGCGCTGAGCAACACAACCTTTAAGTGGTGCGTTCCACAGCATGAATTATAGGGATTCGGTGCTCTAAGTGACACTTGTATACTCATATTTGAACTCATTTATGTTACTCAGAGCACCGAATCCCTATAATTCATGCTGTGGAACGCACCACCTAAAGGTTGTGCTGCTCAGCGCACCTGACCAATAGATGGTGCTATTCCAGCAAATTTTGCCTTAAAGTTATGGAAAAGAGTGAAAAATATTGTTTTATTAACACATGGGCAAGTAAACTATAAAAGTATGTACGGTTAAAAAAAGTATGAAATTAGACTAAAAAAAGAATCGAGAGTCTTAAAAGATTATGTGAAAAGTTACATGGTCTAATCATTTGTACTTCCTCCGTTTTCGAAAGGATGCAACAAAGGGGTATTTTTTGTGAGATATAAAAAATTACTTTGTTGCATCCTTTCGAAAACGGAGGAAGTATTTGGTGAGACGATCGGTTGAGAGATTTAGCTAATTAATAATTGAATAGACAAGCTGACAAGTAATGGACATTAGTTTTCTTCTCATCCTTCTAAACATTTGATAAAGCAAGTGTTTTTCTACTAATTAAAGCAAAATTTGCTTTTTCTAAACTAATTGTTGAGGAAATTATTAAAAAAAAAAAAAAAAAAAAACGAATTTAAATGACTATATTTTTACTTACATTTGTAACAAACTTGGAAGGTTCTCTCTGGTTCTAAGTAGGGAGAAATTTTTAAAAAGAAAAAAGGTGTATCTAAAACTGTGAGGATCATGATTAGCGAATCCTATTCTGATTAGGTACATATTATTGTGTATCGTATTCCATATATTAACATGGATCTTGTTAAAAAGTGTTTCGAGGCATTAGATAGATTAATATATCTTTCAAAATGATATTAATTTTAAGATACATGTCAAAATCTTAAAGTGATTAGTGTATTTTATTTAATTTTAACTTGAATGTTAGCTACCCTAATTTTTATACTCCCTCCGTTTCATTGAATTAGTTACGTTTGGAATATTCAGGACAAGGAAAGAGAAATTTTAAAAGTAAAAAATCATCATCATACACAAGAAAAAACATATTCAATATGGATCTTGTTATATTCGTCTAAATGTGTAGCTTTTCAATATTTACTTTGTATAATTTTTCATTAAGCGTAGTTTGAGATTTTAATGTTTAAACTTTAAAGAAGTGCATCGAAAACTGCGCAAAAAGTAAATGTAACCAACTTAATGAAACGGAGGGAGTACTACTTATTAATGTTCAAGTTTAATACAGTACTTTATACGTACTTTATAGACTCCCTCCGTTCTTCCAAATTGTGGACATTTACTATTTGGGTGGGGATTTAGGAATATTGTTTAATAATGTAAGTGGGGTGTTAAAGTGTAAATAGATTGGAGAGAGAGTGTATAAAAGGGAATAAAATAATAGAAAGTGGGGTAAGTAAGGAAAAGTGGGGTTAAAAAGTGTAAATAAATAGGAGAGAGAATGTCCCAAAATAGAGTAAAGCAATATGTACACAATTTGGAAGAACGTCCAAATGAGTAATATGTACACAATTTGAAAGAACGTCCGAATGAGTAATATGTACACAATTTGGAAGAGCGGAGGGAGTATTAAAAATAAGGTATCTTATGAGTATTTAACTTCAATTGACCTTGATTTCATTTATACATAAGAAAACAAATACTTCCTGTGTACTACTTTTGACTATATCTTGTACTTCCCTCCGTTTCACATTAAGTTGGTTATGTTTGCTTTTTGCACAGTTTCAATGCACTTCTTTAAACATTAATATCTCCAACTATGCTTAATGACAAATTATGCAAAGTGAATATTAAAAAACTACACATTTAGACGAATCTAACAAGATCCATATAAAGTATGTTTTATCTTGTGTATGATGATGATTTCTTACTTTTAAAATTTCTCCCTCCTTATACAGAATATTTCAAGCGTAACTAACTTAATGAAACGGGTGAAGTAATTTATAGACCTATAAGAAAAACTCAACCATATAAAATCATGTTAAATTCATTTATTGTAAAGGCAAAAGCTAAAGGACTCTTTAAAGGCATTTGTTAAGGTAGTTAAATATTAAAAAAATAACTTAAAATAAACACTAATTATATTGAATGTTGTGTTATAGACAAATATTCCAATATTTTTTATCTTTTAGGAAACATATTTATTTTTTACCCAACGCCCCAAAACGTTCGTTAGCAAGATTGATTTGTAAAAGTATCAAATAAAAACAATAATATCTTATACTGAGCATAATAAAATATATAATGATATTTGGAGATTAAAAACGAAAGTAGGAAGAAAGAGTTTATACTTCCTAGTTCATCATGTTATCAACTTTTAATTAGCTTGATCTAACAATAATTGATTTTCCTTACCTCATCCATGATCCAATGGTCCACCATGTAGTAGTTCATAATCAAAACCTAAACCATGAATCTTTTCAAAATTTCCTACCCAATAATTGTTCAAGCAAGATAATTTTCTATAATACTCCTACTTAATTATTGGAAATCAATTGAGTCTTAACTATTCATCAAATGACATGTTACATAGACTTCTCTTGATCATCCACACAATTATTTTAATTATTTGATTGACTAATTTGAAGACTAAAATGATATTCACCTACTATCTTATTCTTATTCTTTGTAATATTCTTACCAAAACATTAAATTATGTTTTTTTCTCAAATAAATTGAACAACACCTTTTAGATGCTAGAAGGTCCTAATTATGCTAATTAGCAAGTATTGTAGTTTACACAATAAATGATAATACTTCCCTTGTCATGCCGTCTATAATTATAAAACTATTCGTATAACTATTTATCATTTATATAATTATATGAGACGTTAAGGTTGTTATATAAAAAGTTATAAATGTCTAATAAAAAAGAATAACCTTTTACTAAAGAGTTTTTCTATTAAACCGTAAGCATGAGACTTCCTCATAAAATAATTAAATTCGTAAGTAGGCATTTATTTAATTCAATAATTGTTGCATATTTTCTTATAACTAACTCTTTCTAAGTTACTAATTGAAGTTTTATTATACTTTCAAACTACCTTTGACATGAAAATTAATTTCTTGACTGACTTTACGAAATTCCTTAATTGATTATTAAGTTTACAGTCATAATATTAATGATCCAGGTTTATATTTGTTGTCCTTTATGATTATTTAATTTGCATGCAAAATAATACACAGGTATCTTCCACCTTACACTTCATTTCTTCCAATTAAAGATGAAGAAAAAGAATATCCAAACATGTTAGGATTATGTAAAGGAGAAAAGAAAAGGAGAAAGATATTGAAGAATATCTTATTCTCACAGCTCAGCTATATTGCCATCTTCACCATTATTATTTGCATCACAGAGAAGCAAAAAATTAGAGATGATCCTCTCAATTTCAACGTTTTCAACATTGCCTTTGAAGTTATAAGGTGATGAGGTTTTTTTAATTACTAGTGTCACTATTTCGCATTAATAAGTTCATTAAGTAAAATAAATTGAATAAAACATGCACTCAATGTCTAAAAACACTATGCTAATTTTCACATTTAGACAAAAATACATAAAAGTCAAGACAAATGAGCAAAAAGTTAAGTGGTTCTCATAATTACATCTTGATTTTAAAAGGTATTAGTTGGACAATCTTCCTTAAAACCCCTTATGGAATTAGTTTTATACATAGGGCACAAACTTTATATATATAGAACCTGAACTTATATAACCTAAATTTATTGGAACTTACCTAAACTTATTGGAATTTATAAGAACTTATTGGAACGTATTATAGCTTATTTTTCAACTCACTAGAACTGAATAGAACTTATTAGAACTTATATAACTTATTGGAACTTATTAGAACTTATTATAATCAGAACTTATCTGAACTTATTGGTAAAGAATTACGGTGAAAAGAACACACTCTTAGTATAATTTAGCAAGGAAAAATAACATATAATTTCTATGGTAATGCAACTTCAACCTCGAATTGTGTTTCCTTCATGAATTTTCATTATCATCCATTACCACTTAAGGGGTCATATAGGATGAGAATGAAAATTTATGAAGAATAGACAATTTGAGGAAATTAATATCACCATGGAAATGACCACTGATTCATTTTCTTACAAATTTACGAGCAAATTGATTCCAATAACGATCATAACCAGCTACCAAACTGGACGTAAGGATTTAGCCGATTGTTTTCAATATAGTAAAAAATCATTAACTCAAGAATATGAGAAACTTAGCTTGAATCAAAAGATGAAATTCAAAGATCTATAATGCATTATTTGTTACAATGAAAAATTGGCAGTGCATATGGAAATGTGGGGTTTTCAACAGGCTACAGCTGTGGAAAGCAATTGAAAGCTGATCCAAAGTGTGTGAATAAATGGTATGGATTTGCTGGAAGTTGGAGTGATGAAGGAAAATTGGTTCTAATCATAGTCATGATATTTGGAAGACTCAAGAAATTCAACTTAAAAGGAGGCAAAGCTTGGAAACTACTCTAAATCAATTTATTGAAAGATTCTTAACTCCTCCCGATATATAATGCTCCTTACTCATAAAAATATTCCCATTTTTGGGTAAATTTATGTGGGTACGGAGTATAATATCGGGTGGAGTTACGAATTATTTATACTATATGTATTGTAACTCGATATTAATCATCGAAATTGTGTGAGTTCAAATTTTATGTAGTCGTAAATCTTTTTCCTTTTTTCTTTAAGAATAGTGAAAAGGTCATGCATAGTTTCTTCCTTTCATGATTTCTTTTCTGTGTGTAAATTATACTCTCTCCGTTTCATTTTAATGTTTCAACTAGCTTTTTGCCCGAGCGATGCTCGGTTTACTAAGAGATTATTTATTGTATATATTTTTAAACGAAAATGTACCTATAAGATGTAGATCTTCAATTTCAAATTTTAAAACCATAATAGTTATTTGAAATCTATTAAATACATTTATAATTCTATAAGATAAGCTATTTCATATTGTTATACTCCGTGTGTCAATTTATCTAAAATTATTGTTTTAAATCAAAAAGAATAGATAAATGCATGAAAAACGTATTAGGAAAGATAAAGATTTTACAAATTTTCAACGTAACCAGATAATATGTGTTATCAATTAAGAGACAATCGATTTAGATTTTGCACGTGTATCGCAGAGGTTGTAGTAGATTTTTTTAGTAGATTTTTTTAAAAACATCATTGGTTTATATTTGTTATCATATGATATAAATTATGTGAAGACCTTCAAAAAAGTTGTAACTATATATTAATCATACATGTTTTATAACTAACAAAATTTCAACCTACTTTTTATTGTTATGTTATTGTTATGACAATTTTATTTTTATGTCAAAGAATTAAACACA

>BsHKT1;2

ACTCACTTATACAACTCAGCTTTCTCACAAGAAAAGAAAAGAAAAAAAAAAAGAAGCTAATCATAGACAAATTATTATTATTATTATTATTACACAAATCCAAAGTATGGAGCTTCAACTGTATCTCCTTAAAATCATGGAGAAATACTTAGCTCTTTTGCATGAAAACTCAGACAAAATCAAAGTTTTCTTCCAAAAAAAGGTGTCACCATTTTTTAGTCATGGTTTTGAGTACTTGTTATTCCAAATCAGCCCATATTGGCATCATCTATTCTACTACATCCTAGTGTCTCTTCTTGGGTACATCTCATTGAAGGGCACAAAACAGAGTTATTCATCACCAAAAAAAGCTATTTACAACCCACAACACCATGACCTTGATCTCTTCTTCACCTCGGTTTCTGCCACAACAATTTCAAGCATGTCCACCATTGAAATGGAGAAGTTCTCGAATGCCCAACTTATGGTTATCATACTCTTGATGCTTTCAGGGGGTGAAGTCTTCCTCTCTTTGCTCGGCCTCCAAATCCGAAAGCTTAAACATAAGAAAAGAGCAAGAAACCATCTCTTAAACCCAAACCCTGCTTCACAAGAAGAAGGTATGAAGTATAGGTCATTAAGGGCACTTAATCATGTGGTTTTAGGGTACCTTGTAGTTTCACATATAATAGGGTATAGTTTATTATCCCTATATATAAGCATTGATTCAAGTGCAAGTAATGTACTTGAAACGAAAAAGCTTGAAATTCACCTATTCTCTATTTTTACAACTGTTTCTACATTTGCAAATTGTGGGTTTATACCAACAAATGAGAATATGGTAGTATTCAAGAGGAATTCAGGGTTTCTTTTGATTTTGATACCTCAAATTTTGATGGGAAATAAGCTTTATCCTTGTTGTTTGAGGTTGGTTATATGGGTACTTGAAAGACTTACTAAGAAAGAGGAGTATAGTTATTTGTTGAAGAATCATGAAGAATTGGGATATGGACTTTTGACATCAAATTATAAGGCTTTTCTTTTAGGGATAACTTCAATTGGATTGGTTATAGTTCAGTTTGTGGTTTTTTCTATATTGGAATGGAATTCAGTGGTATTACAAGGACTCAGTTTATATCAGAAAATTGTTGGATCTCTATTTCAAACTGTGAATTCAAGGCATAGTGGAGAGTCTATTCTTGATATTTCCCAAGTTTCACCTGCAACTATGCTGCTCTTTGTTGTCATGATGTGAGTCTCTTCATCCCTCTTTATATTTGCTTCTTCTTCAATTCTTATGTTTGTGGTCCATCATGTCTATTCCCTTTCTTAAATTCTATGCCGTACAATTCATAAATCATTATGAAAAACAAAAAAGAAATAACAACAGCTCGTTTGGTTGATGGTAATAATGAACACGGATTGAAGAACGTGTAATTCTGTATAGAGACATGCTAATGTGGATGAGAATGATTGTCCACCCACATAATTGTGATATCTTTCACTATTTTTCCTTGTTACCATTCATTACCACTTTAGAGATGCATTATATTAAGAAAGAATGCAATTTATAATGAAAAACACATACATCTTATTTTAGATGAGGCTTTATTACCATAGTAAAACGAGCATGGAATATTCTATAATTTATTAATTTTATCAAAAGGGTCAAAAAAGTTTAAGGGTCGTACATTATAAATAGTAAAAATTTATCTGACTGTTTTAGTGATTATGCAATTTATTCATTACGTCCTTGACTTTACTGCCAATTATCAATATATCATAGTACTTACAAAAATAGAGAATGTATACTTTAAATTACAGTATCAAAAGCAACTGAAAGTTAAGTAAAAGGATAGCTTGATTGAGGTAGTTAAAAAGTTCGAAAGAGTGTTAAAAGGGTAATTTTTTTAATTCAAAATGGTGTTAAGTAAGTTGCAAAGATATTGGATTATCGATATTCTAATCTTATCAACTTAATTATCGTGCATAGGTGGGATGCTCTTAGTGTTTGCTATCAATCGTACTCGGATCATTAGATGATGCACTTTTTTCACACGTTATTATTTTTTATTTTTATTTTTATTTTTATTTTCATATTCATCTTTATTTTTATTTTTTACAACTTTACAAGTGTGATTGAAGGTGGTTTTGCGGGACCAAATTAGCGAAACTAGTTGAAATTGAAACCAACAAAAGTTTTACGTGCCACCGGTTCACGGAATCCATGCATGAAATGTTGTTTTCCTGCCCTCCAAAATATAAAAATAAAAACAAATTAATTTACACCTAAGAAAAGTTGTTTTTGCAACGTACTCTCTTTGTCAACCAAATACAATGAATAAGATACTAGATTTTTTTTTCTTTTTGTAGGAGAGCCGACTTGTTTTTATGTAAATAAATCTAATTAATGAAGATTCAAATTTTAATTTGAGAATCTTAAGAAATGACAGTTATAACAACAATTAATTAATTAATTATTTCAATTAGTACAGTCATAGTTTAGTGAGTATCAAATTTGGTTAAGTTATGATAATTTTGAACATGCTTTTCATATATAAATATGCAAATATAAAGTTATTTGAGATTTTGTTAGATTTGACTGAATGTGTATAATCTATTTATCACTTTTTTATAAACTTTTAATAATTTATATAGAAGATATTAATAATCAAAGTTATAATTTACCGTCTTTGAAAAGGCGGATAGAAAAAACTTTGTAAAAAGGAAAGATTATGATATATTATTGTAATACTACAACAATATAAATAAAAGACAACATATCAGCATCTTATAATTGGTTTCACACTAATTTTGTCTTATCCAATAATGCTTGGATCTTTATATTGTTCTTCTTTCGAAGATCGGCATGATCAACGTAATTATCTCAAGCAATAATGTGTCAAAAAAAAGTAATTATCTGAAGCATCATATATGTACTGATATATGTACTACACTATAATTCAACCTCACAATAATAACAAAACTAATTAATCATTAAAGTGACATGCTAATGTCTTACAAACATCAATTATGAAAATTTTCAATTTTATACTCAAAATAAATTCTTTTTTAAAGCTGAATGAAAACAATTTTAATATGAACTATAATTTTTATGATAACCACAAAAAAAAAAAAAAAAAGAAAAAAAATAGTATTTATGTCTAGAAGAAAATAAGTTCCAAGGACTCACGATTTTGCCAGTAGATATACTTCCTCTGCTTTTTTTTCCTCGTAATGTAGGTCATTTTAGGAAGTTTTACAATGTTACTAAATTTAGTAACTTTATTTTATGAAAGTACTATTATGACCTTTAAATTACACAATTAATTCCCATTTCTTTTGTATATTTACCTTTATAGTAACAGTAAGGGCAACTTCGACATTTTGATTTATTCTCTGACCAACTCAAAAAACTAGTAACTAGTACTAAAAATAGAGACCTACATTACAAGGGAATAGGGGATATATATATATATATATATATATATACACACACACACACTTCCTCCGCTCCATATTACTTGTAACAGAGCCATTCGACACGTATTTTGAGGAAGAGATTTAAAGATATAAACGAGAAAATTAGTCAAATTTTTTTTAACACCTTTAACATATAATTTCCCACTTGCTTATGTATAAATATTTTTAATTAGTTTGATCGTTTATATGGCCAATTTTCTTCTCCAAAATACGTGCCAAAGAAGCAGTTGGGAGTAATATGCGGAGGAAGTATTATATATATATATATTTATACTCGTAATATACTTGAAGAAAACAGAAAAATGTGCTCGTGTATAAATAAAAATATTAATATACACTTCTTGATTGCCACATGATTGCACGTTCTACAGGGCAGATGAAGCTACTAAAGACTAATATATGGCTCTATCACATTATCTGTCATTTGGGAGGACCACTTGATTTTTTGTTGTACCATCAAAATAAATTGATTGGTATGAATGAAATAATAAATGAATAATTTACCTTAGAAATCATTTATATTTTGCTTATGTTGGCCTCTCTTTTGACCTCAACCTACATCCTATGACCTACTACTACCTAGCCTTTTCTCACCCATTCAATTTCTAGGTATTGTTTTTCCAAATTAAGTAAGACTTTTGGAGTTATTTGGGGGAGGGGTCTTTCTATACTTAAGAAGAACTAGGTATTACTTGAGCAATCAAGCAATGATTATTAGTTTAAATCAATATGAAAATAGTTAAATAGCTACATTAAACTCTAGTTTTCAACTTTACAGTCTAGTTTCTAATGGATACCTAATAAAATGTAACAACGTATGTTTATCAAGGTTTTTTGATTAGAACGGAGATACTTGTTAGTGGTTTTTCTGTAATATAATTAAAGAACTTGTATTGACTTGAACAATAAACTAAGTCAGTTAGGGGGGAAAAACAGCTAACTTAGGCTTTGACACTAGAAGTATCTTTTTTCTTTTCTTCCACACTATTTTTGTCAATTTTAGAGCCCCAACACCAAAATCTGGCTACATTCATGTACAAAAAGAATTATTATTCATTCATCCGTCTCTCATTCTTTTATTCTCTTTAAATTATTAATATTTATATGTTGTTCCAAGAATATTAATTTAACATCTTTTCGATCATGTCTTTCAACACTAGATGAACATAATAGTTTATTCAATAATCTTTTTATTTGGAATCAATTGTCATATTTGACTTCTTATAGTAAAAGATGACAAGCTTAATATAATAATGTTCTTATGTTCCATACAGAATATAGTATAGATACAACTTTATTGACAGTGTGCATGACTAATGTTTCAGCTTGCATAGACATATATTTATGGGCATAAATGTGAAACTGAAATTTAGTTAGTAATAATATATACTTCCTCCATTTTTTAATAACTGCAACAAATGAGAATATTTGTGAGAGATAAAAAGTTCATTTGTTACAAAAATATGTGTATTGACGAAAAAATTGAAATGAAAATGGCAGGTACCTTCCATCCCAAACGACATTCGTACCAATATGCTACGACAAAGAATCAACATTAATAGAGAACAAGAGCCATCATCGAAGCAAAAAGCAAAAGAGTAGTTTCATAGAGAATCTCAAATTCTCACCTCTTTCCTACTTGTCAATCTTTGTCATGCTTATTTGCATCACAGAAAGCAGATATTTGAAAGAGGATCCTCTTAATTTTAGCGTCTTCAACATTATTGTCGAAGTTGTAAGGTATTCCTTCTTAGCTTATTCTTATCTCTTCTCTATCAATCAACCAACATAATATATTCCATCATCTTCATCTTAATCGATTTCATATTTTTTGATCCGATAATCCTCATTTGTTATACAAGATTGGATGAATAA

>BsHKT1;3

TTATTTTTCTACATTTATATGTGGATTCTACTTTGCTCTCATTAGCATGAGATATGAGGTCTTATTGAACACACACATAATAAACTTTCAAAAGATTCAAAATGTATGATCATTCTTATATCAAAACTAAATAAAACTACTCATTGTTATGGTATCACATAAGATGCATGTCCATGCCTAGAAATCTACTAACACAAAATACAGTTAAAAATATACCCAGTCCATATTCTAATACCCTTAGACTATTATACTCATCTCTACAAACTCATGCAAATTATCAACATTATATAAATTAATAAGCTGTAAAATTAGATGAAGCAAATATTTACATTAACAAAATGAATAGTGAAAAGAGAGAAAAAACTCACATCATGATAGTGAATAGCACCAAGACTGCTTGAGAAAGTTGAGAGATATCAACAACAGATTCTCCACTATGCCTTGTATTTGCGGTTTGAAATAAAGAAGCCACAAACTTCTCATAAGAGCTCATTCCCTCCATAACTTGAGAGCTCCAATCCATACCACAAAATACTACAATTTGTAGCACCAAAAACACTATTGTAGTACCACCCAAATACCAACATTTTTTACTTGACATTAAATGACCATATTCTAACTCCTCATAATTCTTCAATATGTAATTAAACTCTTTCTTTCTACTAAGCTTCTCTAACACCCATATAACTAGCCTCAGGAACAATGGATACATGGTATTTCCGACGAAAGTGTAAGGAAGAAGAATCAAAAGAAGGCCAGGATTGTTCATCCTAAAGATCATCATGTTTTCATTTGTAGGTGCAAAACCACAACTAGAAAATGTTGAAACTACCATGAAGAAAGAAAATGTTTGTAATACAAGTCCTTTATTATTAAGCACATTTAGAGCACTAGGAGTTAGGCTCATGTACATGGTTATCAAAGTTGATCCTACTAAATGTACAATAATTATGTATCCTAGGACCACATAACCTAATAATTTAATTGACTTGTTCTTATTATACTTAAATGAAGAAGAATTATTACTACTAGTAGTAATAATATTACTAATTATCTCATGATTATTGTTATTATCATCAATAGTTGAAATAGGAGGTTTGATCATGCCTAACTCTATAGAATCTATTGAGCATGAACTCTCTAATTTCGGGTTTTGGATAGAAGGAAATTCACAGCTTCGAAGATGGAGGCCGAGCATGGAGGTGAAGACTTCACCTCCTAATAGCATTAGAATAGTCATAACAATTAGTTGATCGTTAGAAAATACCTCCATTTCAACTGTAGTCATGCTTGAAGCCGTTGCAGCTGAAACGGAGGTGAAAAAAAGATCAAGATTGCTCGGAGTTTCGCTAGTTCTTGTCTTTGAGACCTTCAAAGACAAAAAACCAGCTAATGAAAGAACAAGAAAATAGCAAAGATGAAGAGAAAATGGGTTTATAAGAGGGATTATGTAGTGATGAAAAATTGTTGTTATTGAGGCTTTCATGAGTGAATAACAAGTGATGAAGAATGATTTTTTGGGGAAATAATCTTTACAAAATTGCCTCTAATTTATTGCGTAAAGAAGAGTAGTAATTCATGATATTCATTATGGAAGCCT
