# Supplementary material for: Genome-wide identification of a novel Na+ transporter from Bienertia sinuspersici and overexpression of BsHKT1;2 improved salt tolerance in Brassica rapa
Source: Front Plant Sci. 2023 Dec 12;14:1302315. doi: 10.3389/fpls.2023.1302315 (PMC10773568; doi:10.3389/fpls.2023.1302315)
Supplement: Supplementary file 1 [file DataSheet_1.zip › Supplementary File 3.DOCX]

**Supplementary file 3. Coding sequences of BsHKTs.**

>AtHKT1;1

ATGGACAGAGTGGTGGCAAAAATAGCAAAAATCCGTTCGCAGCTTACTAAATTACGTTCA

CTATTCTTCCTTTACTTCATCTACTTCTTGTTCTTCTCCTTTTTAGGGTTTTTGGCACTC

AAGATCACAAAGCCAAGAACCACTTCACGTCCTCATGACTTTGACCTTTTCTTCACTTCT

GTCTCTGCCATCACCGTCTCTTCCATGTCTACCGTCGACATGGAAGTCTTCTCCAACACC

CAACTTATCTTCCTCACTATCCTCATGTTCCTCGGTGGCGAAATCTTCACCTCCTTTCTC

AACCTCTACGTCTCCTATTTCACCAAGTTCGTCTTCCCTCATAACAAGATTAGACATATT

TTGGGATCTTATAATTCGGACAGTTCCATCGAGGATCGCTGTGACGTTGAGACTGTTACT

GATTATCGCGAGGGTCTTATCAAGATCGATGAAAGGGCATCTAAGTGCTTGTACTCGGTG

GTTCTTAGTTACCATCTTGTTACTAACCTAGTTGGCTCTGTGTTGCTTCTTGTGTACGTA

AATTTTGTTAAAACGGCGAGAGATGTTCTTAGTTCCAAAGAAATCTCACCTCTCACTTTC

TCCGTCTTCACAACTGTTTCCACGTTTGCAAACTGCGGATTTGTCCCCACGAATGAGAAC

ATGATCATCTTTCGCAAGAACTCTGGTCTCATCTGGCTCCTAATCCCTCAAGTACTGATG

GGAAACACTTTGTTCCCTTGCTTCTTGGTTTTGCTCATATGGGGACTTTATAAGATCACA

AAGCGTGACGAGTATGGTTACATTCTCAAGAACCACAATAAGATGGGATACTCTCATCTA

CTCTCGGTTCGTCTATGTGTTCTTCTTGGAGTGACGGTGCTAGGGTTTCTGATAATACAG

CTTCTTTTCTTCTGCGCCTTTGAATGGACCTCTGAGTCTCTAGAAGGAATGAGTTCGTAC

GAGAAGTTGGTTGGATCGTTGTTTCAAGTGGTGAATTCGCGACACACCGGAGAAACTATA

GTAGACCTCTCTACACTTTCCCCAGCTATCTTGGTACTCTTTATTCTTATGATGTATCTT

CCTCCATACACTTTATTTATGCCGTTGACGGAACAAAAGACGATAGAGAAAGAAGGAGGA

GATGATGATTCCGAAAATGGAAAGAAAGTTAAAAAGAGTGGACTCATCGTGTCACAACTT

TCCTTTTTGACGATATGTATCTTTCTCATTTCAATCACCGAAAGGCAAAATCTACAACGT

GATCCGATAAATTTCAACGTCCTTAACATCACTCTCGAAGTTATCAGTGCATATGGAAAC

GTTGGTTTCACTACCGGGTACAGCTGTGAACGGCGTGTGGACATCAGCGATGGTGGCTGC

AAAGACGCGAGTTATGGGTTTGCAGGACGATGGAGTCCAATGGGAAAATTCGTACTAATA

ATAGTAATGTTTTATGGTAGGTTTAAGCAGTTCACAGCCAAATCTGGCCGCGCATGGATT

CTTTACCCCTCGTCTTCC

>BsHKT1;1

ATGTTGAATTTCAACTTTATTGTAGAAAATTGTAAACAATTTTATACTTCTTTTTGTCTA

CTTTTTGCCTATATTTTTACATCCTTATATTGGTTATCCTCAAAAATCTATGATTTTATC

ATCATTTATGTTAGCCACTTTATAATTGAACTATGCTACTTTATCCTTGTATCTTCTTTT

GGATTCTTGTTTTTAAAAACCCTAAATCCAAGATCAACCCATAATAATCACCCAATAATT

AATGATTTAGATCTATTCTTCACCTCAGTTTCAGCCACAACAGTTTCAAGCATGTCAACC

CTAGAAATGGAGGTATTCTCAAATTCCCAACTAATTGTTTTAACCATTTTAATGTTCATA

GGAGGTGAGGTCTTTACCTCCATGGTAGGTCTCCATTTTTCGGCCTCGAAACTTGTATAT

ACACCCTTACATTCAAGAAGTAGGGTTAATTCAGTTGCTAGCTTACCACTTCCTTCTGAA

GGTATTGAGTTAGGAATCATTATCCCATCATCAAATGAAGCTTCTTCAATTGAAAAAACA

AAATCAGAAATAGATTTCCTCATAAAATCTAAATCAATTAGGGTTTTAGGTTTCATAGTT

TTGTCTTACTTATTCATAGTTCATTTCCTAGGAATTTCCATGGTATTAGCATACATTAAT

ACTATCCCAAATGCCAAAAATGTTCTTGACAAAAAAGGTCTTAAAACATTCACTTTTTCA

ATTTTTACAATTGTTTCAACTTTTGCTAGTTGTGGTTTCATCCCTACTAATGAAAACATG

CAAGTTTTTAGCAAAAACTCTGGCCTTTTATTGATTTTAATCCCTCAAATTCTACTTGGA

AACACATTATTTCCTTCATTTCTTCGATTTTCGATATGGGTATTAGGAAAATTTGCCAAA

AAAGACGAAACTAAATTTCTAATGAGAAATTCAAAGGAAATTGGGTACCATCATTTGCTT

CCTAGCAAACACTCAAAGTTTTTAGTAGTAACAGTTTTGGGGTTTATTTTGGTGCAATTT

ATAATGTTTAGTTCAATGGAATGGAATATTGAAGGATTAGATGGACATAATATATACCAA

AAATTAGTGGGAATGTTATTTCAATGTGTTAATTCAAGACATACAGGTGAAAGCATTGTT

GATCTTTCCTCAATTGCATCAGCTATGTTGGTCATGTTCATCGTTATGATGTATCTTCCA

CCTTACACTTCATTTCTTCCAATTAAAGATGAAGAAAAAGAATATCCAAACATGTTAGGA

TTATGTAAAGGAGAAAAGAAAAGGAGAAAGATATTGAAGAATATCTTATTCTCACAGCTC

AGCTATATTGCCATCTTCACCATTATTATTTGCATCACAGAGAAGCAAAAAATTAGAGAT

GATCCTCTCAATTTCAACGTTTTCAACATTGCCTTTGAAGTTATAAGTGCATATGGAAAT

GTGGGGTTTTCAACAGGCTACAGCTGTGGAAAGCAATTGAAAGCTGATCCAAAGTGTGTG

AATAAATGGTATGGATTTGCTGGAAGTTGGAGTGATGAAGGAAAATTGGTTCTAATCATA

GTCATGATATTTGGAAGACTCAAGAAATTCAACTTAAAAGGAGGCAAAGCTTGGAAACTA

CTC

>BsHKT1;2

ATGGAGCTTCAACTGTATCTCCTTAAAATCATGGAGAAATACTTAGCTCTTTTGCATGAA

AACTCAGACAAAATCAAAGTTTTCTTCCAAAAAAAGGTGTCACCATTTTTTAGTCATGGT

TTTGAGTACTTGTTATTCCAAATCAGCCCATATTGGCATCATCTATTCTACTACATCCTA

GTGTCTCTTCTTGGGTACATCTCATTGAAGGGCACAAAACAGAGTTATTCATCACCAAAA

AAAGCTATTTACAACCCACAACACCATGACCTTGATCTCTTCTTCACCTCGGTTTCTGCC

ACAACAATTTCAAGCATGTCCACCATTGAAATGGAGAAGTTCTCGAATGCCCAACTTATG

GTTATCATACTCTTGATGCTTTCAGGGGGTGAAGTCTTCCTCTCTTTGCTCGGCCTCCAA

ATCCGAAAGCTTAAACATAAGAAAAGAGCAAGAAACCATCTCTTAAACCCAAACCCTGCT

TCACAAGAAGAAGGTATGAAGTATAGGTCATTAAGGGCACTTAATCATGTGGTTTTAGGG

TACCTTGTAGTTTCACATATAATAGGGTATAGTTTATTATCCCTATATATAAGCATTGAT

TCAAGTGCAAGTAATGTACTTGAAACGAAAAAGCTTGAAATTCACCTATTCTCTATTTTT

ACAACTGTTTCTACATTTGCAAATTGTGGGTTTATACCAACAAATGAGAATATGGTAGTA

TTCAAGAGGAATTCAGGGTTTCTTTTGATTTTGATACCTCAAATTTTGATGGGAAATAAG

CTTTATCCTTGTTGTTTGAGGTTGGTTATATGGGTACTTGAAAGACTTACTAAGAAAGAG

GAGTATAGTTATTTGTTGAAGAATCATGAAGAATTGGGATATGGACTTTTGACATCAAAT

TATAAGGCTTTTCTTTTAGGGATAACTTCAATTGGATTGGTTATAGTTCAGTTTGTGGTT

TTTTCTATATTGGAATGGAATTCAGTGGTATTACAAGGACTCAGTTTATATCAGAAAATT

GTTGGATCTCTATTTCAAACTGTGAATTCAAGGCATAGTGGAGAGTCTATTCTTGATATT

TCCCAAGTTTCACCTGCAACTATGCTGCTCTTTGTTGTCATGATAGAACAAGAGCCATCA

TCGAAGCAAAAAGCAAAAGAGTAG

>BsHKT1;3

ATGAAAGCCTCAATAACAACAATTTTTCATCACTACATAATCCCTCTTATAAACCCATTT

TCTCTTCATCTTTGCTATTTTCTTGTTCTTTCATTAGCTGGTTTTTTGTCTTTGAAGGTC

TCAAAGACAAGAACTAGCGAAACTCCGAGCAATCTTGATCTTTTTTTCACCTCCGTTTCA

GCTGCAACGGCTTCAAGCATGACTACAGTTGAAATGGAGGTATTTTCTAACGATCAACTA

ATTGTTATGACTATTCTAATGCTATTAGGAGGTGAAGTCTTCACCTCCATGCTCGGCCTC

CATCTTCGAAGCTGTGAATTTCCTTCTATCCAAAACCCGAAATTAGAGAGTTCATGCTCA

ATAGATTCTATAGAGTTAGGCATGATCAAACCTCCTATTTCAACTATTGATGATAATAAC

AATAATCATGAGATAATTAGTAATATTATTACTACTAGTAGTAATAATTCTTCTTCATTT

AAGTATAATAAGAACAAGTCAATTAAATTATTAGGTTATGTGGTCCTAGGATACATAATT

ATTGTACATTTAGTAGGATCAACTTTGATAACCATGTACATGAGCCTAACTCCTAGTGCT

CTAAATGTGCTTAATAATAAAGGACTTGTATTACAAACATTTTCTTTCTTCATGGTAGTT

TCAACATTTTCTAGTTGTGGTTTTGCACCTACAAATGAAAACATGATGATCTTTAGGATG

AACAATCCTGGCCTTCTTTTGATTCTTCTTCCTTACACTTTCGTCGGAAATACCATGTAT

CCATTGTTCCTGAGGCTAGTTATATGGGTGTTAGAGAAGCTTAGTAGAAAGAAAGAGTTT

AATTACATATTGAAGAATTATGAGGAGTTAGAATATGGTCATTTAATGTCAAGTAAAAAA

TGTTGGTATTTGGGTGGTACTACAATAGTGTTTTTGGTGCTACAAATTGTAGTATTTTGT

GGTATGGATTGGAGCTCTCAAGTTATGGAGGGAATGAGCTCTTATGAGAAGTTTGTGGCT

TCTTTATTTCAAACCGCAAATACAAGGCATAGTGGAGAATCTGTTGTTGATATCTCTCAA

CTTTCTCAAGCAGTCTTGGTGCTATTCACTATCATGATGTGA
