# Supplementary material for: Genome-wide identification of a novel Na+ transporter from Bienertia sinuspersici and overexpression of BsHKT1;2 improved salt tolerance in Brassica rapa
Source: Front Plant Sci. 2023 Dec 12;14:1302315. doi: 10.3389/fpls.2023.1302315 (PMC10773568; doi:10.3389/fpls.2023.1302315)
Supplement: Supplementary file 1 [file DataSheet_1.zip › Supplementary File 4.DOCX]

**Supplementary file 4. Amino acid sequences of AtHKT and BsHKTs.**

>NP_567354.1 (AtHKT1)

MDRVVAKIAKIRSQLTKLRSLFFLYFIYFLFFSFLGFLALKITKPRTTSRPHDFDLFFTSVSAITVSSMS

TVDMEVFSNTQLIFLTILMFLGGEIFTSFLNLYVSYFTKFVFPHNKIRHILGSYNSDSSIEDRCDVETVT

DYREGLIKIDERASKCLYSVVLSYHLVTNLVGSVLLLVYVNFVKTARDVLSSKEISPLTFSVFTTVSTFA

NCGFVPTNENMIIFRKNSGLIWLLIPQVLMGNTLFPCFLVLLIWGLYKITKRDEYGYILKNHNKMGYSHL

LSVRLCVLLGVTVLGFLIIQLLFFCAFEWTSESLEGMSSYEKLVGSLFQVVNSRHTGETIVDLSTLSPAI

LVLFILMMYLPPYTLFMPLTEQKTIEKEGGDDDSENGKKVKKSGLIVSQLSFLTICIFLISITERQNLQR

DPINFNVLNITLEVISAYGNVGFTTGYSCERRVDISDGGCKDASYGFAGRWSPMGKFVLIIVMFYGRFKQ

FTAKSGRAWILYPSSS

>Bsv0100-00034511-RA (BsHKT1;1)

MLNFNFIVENCKQFYTSFCLLFAYIFTSLYWLSSKIYDFIIIYVSHFIIELCYFILVSSF

GFLFLKTLNPRSTHNNHPIINDLDLFFTSVSATTVSSMSTLEMEVFSNSQLIVLTILMFI

GGEVFTSMVGLHFSASKLVYTPLHSRSRVNSVASLPLPSEGIELGIIIPSSNEASSIEKT

KSEIDFLIKSKSIRVLGFIVLSYLFIVHFLGISMVLAYINTIPNAKNVLDKKGLKTFTFS

IFTIVSTFASCGFIPTNENMQVFSKNSGLLLILIPQILLGNTLFPSFLRFSIWVLGKFAK

KDETKFLMRNSKEIGYHHLLPSKHSKFLVVTVLGFILVQFIMFSSMEWNIEGLDGHNIYQ

KLVGMLFQCVNSRHTGESIVDLSSIASAMLVMFIVMMYLPPYTSFLPIKDEEKEYPNMLG

LCKGEKKRRKILKNILFSQLSYIAIFTIIICITEKQKIRDDPLNFNVFNIAFEVISAYGN

VGFSTGYSCGKQLKADPKCVNKWYGFAGSWSDEGKLVLIIVMIFGRLKKFNLKGGKAWKL

L

>Bsv0100-00016847-RA (BsHKT1;2)

MELQLYLLKIMEKYLALLHENSDKIKVFFQKKVSPFFSHGFEYLLFQISPYWHHLFYYIL

VSLLGYISLKGTKQSYSSPKKAIYNPQHHDLDLFFTSVSATTISSMSTIEMEKFSNAQLM

VIILLMLSGGEVFLSLLGLQIRKLKHKKRARNHLLNPNPASQEEGMKYRSLRALNHVVLG

YLVVSHIIGYSLLSLYISIDSSASNVLETKKLEIHLFSIFTTVSTFANCGFIPTNENMVV

FKRNSGFLLILIPQILMGNKLYPCCLRLVIWVLERLTKKEEYSYLLKNHEELGYGLLTSN

YKAFLLGITSIGLVIVQFVVFSILEWNSVVLQGLSLYQKIVGSLFQTVNSRHSGESILDI

SQVSPATMLLFVVMIEQEPSSKQKAKE

>Bsv0100-00016849-RA (BsHKT1;3)

MKASITTIFHHYIIPLINPFSLHLCYFLVLSLAGFLSLKVSKTRTSETPSNLDLFFTSVS

AATASSMTTVEMEVFSNDQLIVMTILMLLGGEVFTSMLGLHLRSCEFPSIQNPKLESSCS

IDSIEYNKNKSIKLLGYVVLGYIIIVHLVGSTLITMYMSLTPSALNVLNNKGLVLQTFSF

FMVVSTFSSCGFAPTNENMMIFRMNNPGLLLILLPYTFVGNTMYPLFLRLVIWVLEKLSR

KKEFNYILKNYEELEYGHLMSSKKCWYLGGTTIVFLVLQIVVFCGMDWSSQVMEGMSSYE

KFVASLFQTANTRHSGESVVDISQLSQAVLVLFTIMM
